# Environmental predictors of habitat suitability and occurrence of cetaceans in the western North Atlantic Ocean

**DOI:** 10.1038/s41598-019-42288-6

**Published:** 2019-04-09

**Authors:** Samuel Chavez-Rosales, Debra L. Palka, Lance P. Garrison, Elizabeth A. Josephson

**Affiliations:** 1 Integrated Statistics, 16 Sumner Street, Woods Hole, MA 02543 USA; 2 NOAA Northeast Fisheries Science Center, 166 Water Street, Woods Hole, MA 02543 USA; 3 NOAA Southeast Fisheries Science Center, 75 Virginia Beach Drive, Miami, FL 33149 USA

## Abstract

The objective of this study was to identify the main environmental covariates related to the abundance of 17 cetacean species/groups in the western North Atlantic Ocean based on generalized additive models, to establish a current habitat suitability baseline, and to estimate abundance that incorporates habitat characteristics. Habitat models were developed from dedicated sighting survey data collected by NOAA- Northeast and Southeast Fisheries Science Centers during July 2010 to August 2013. A group of 7 static physiographic characteristics and 9 dynamic environmental covariates were included in the models. For the small cetacean models, the explained deviance ranged from 16% to 69%. For the large whale models, the explained deviance ranged from 32% to 52.5%. Latitude, sea surface temperature, bottom temperature, primary productivity and distance to the coast were the most common covariates included and their individual contribution to the deviance explained ranged from 5.9% to 18.5%. The habitat-density models were used to produce seasonal average abundance estimates and habitat suitability maps that provided a good correspondence with observed sighting locations and historical sightings for each species in the study area. Thus, these models, maps and abundance estimates established a current habitat characterization of cetacean species in these waters and have the potential to be used to support management decisions and conservation measures in a marine spatial planning context.

## Introduction

The Northeastern coast of the United States is one of the most populated portions of the country and supports some of the highest intensity of shipping, fishing and marine development in the nation. Not only has ocean use increased dramatically during the past 40 years, but the underlying marine ecosystem has also experienced changes in ocean water temperatures^1^.

A number of cetacean species listed in the Endangered Species Act (ESA) and protected by the Marine Mammal Protection Act (MMPA) are subject to these environmental and anthropogenic pressures^2^. Cetaceans play important roles in the marine ecosystems as predators whose dynamics are associated with the mid-trophic levels through trophic linkage^3^. Consequently, these species not only affect entire food webs, but are also affected by the dynamics of the physical and biological environment^4,5^. Detailed current knowledge of the distributions of cetaceans and their suitable habitat is important for the effective management and conservation not only of cetacean species but also of entire marine ecosystems^3^. This is particularly important given the rapidly changing oceanic environment in the Northwest Atlantic Ocean off the U.S.^6^ and the increasing demands for energy production that promoted the development of renewable energy areas on the outer continental shelf^7^.

Results from habitat suitability models, their underlying spatial-temporal density distribution maps and the relationships between habitat features and density patterns are a cornerstone to support conservation and management. For example, they can be used to predict and monitor species’ response to changes in the climate and anthropogenic impacts^8,9^, and generate abundance estimates that support conservation and management^10,11^. In addition, these models have the potential to identify priority conservation areas, and diversity hot or cold-spots^12^. Several U.S. federal agencies require information about spatial-temporal density, habitat, abundance, population size and predictive models for marine protected species to support their environmental compliance documentation related to the National Environmental Policy Act (NEPA), MMPA and ESA. For example, the U.S. Department of Energy and the U.S. Bureau of Ocean Energy Management are working closely with several states, to establish offshore renewable energy developments within 50 miles of the eastern U.S. coastline on the outer continental shelf^7^. Other examples are regulations under the MMPA to govern the unintentional taking of marine mammal incidental to training and testing activities conducted by the U.S. Navy^13^. In both of these examples, identifying suitable habitat would help constituents to determine how to minimize human and cetacean interactions and an important component of their respective Environmental Impact Statements.

The objective of this study was to provide this background information for the above conservation and management needs; specifically, to use generalized additive models to establish a current habitat suitability description for cetacean species, to identify the main environmental covariates related to cetacean distribution, and to estimate abundance accounting for habitat relationships. Further, and example application of these models in conservation and management issues is discussed.

## Results

### Habitat models

**Figure 1:**
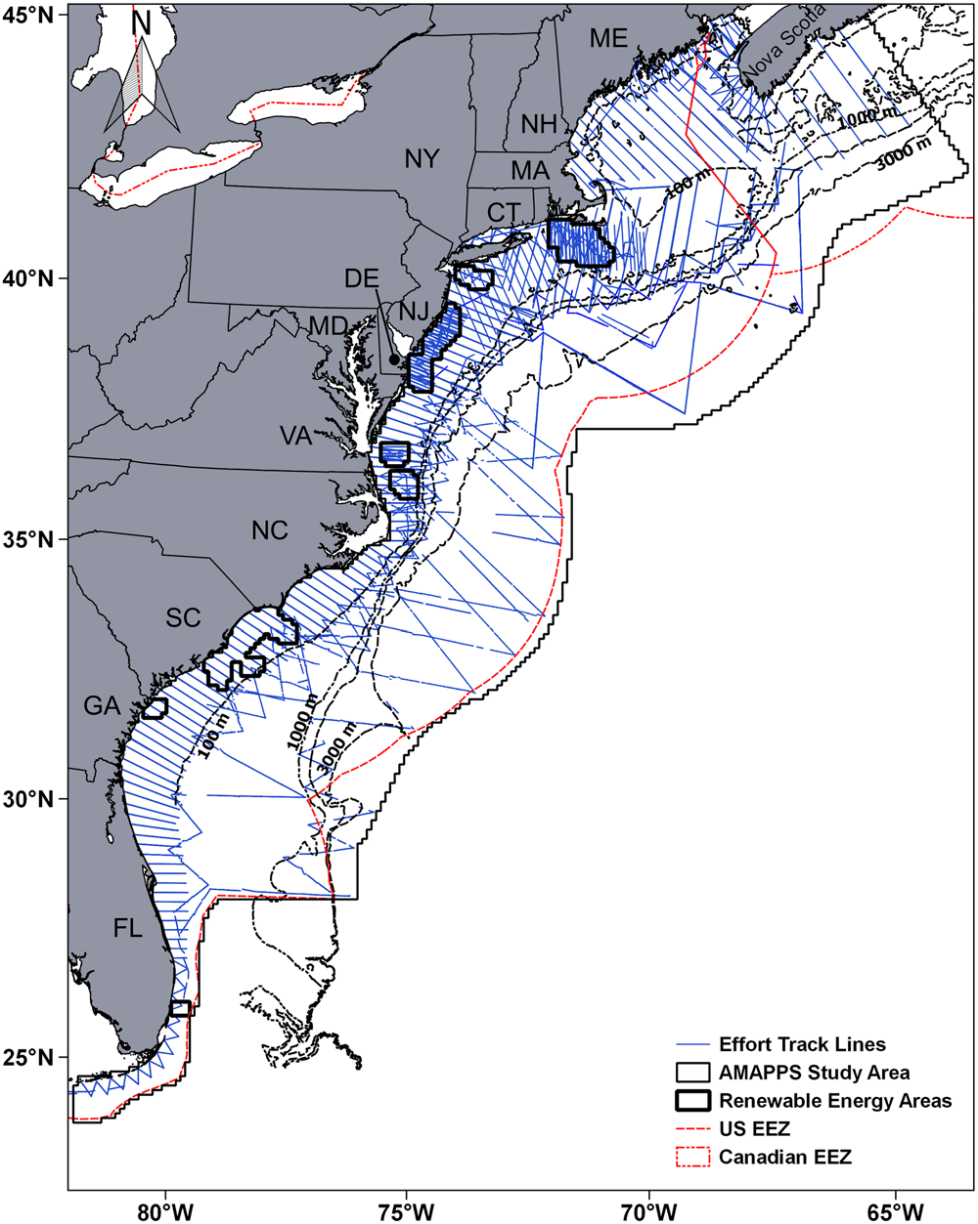
Effort track lines in the AMAPPS study area during 2010–2013, most track lines were surveyed multiple times by shipboard and aerial surveys. Renewable energy areas include a 10 km buffer zone.

A total of 103,395 km of track line was divided into 13,792 spatial-temporal cells (3,329 for spring, 5,978 for summer, 3,237 for fall and 1,248 for winter) that covered offshore and coastal habitats including the renewable energy areas (Fig. 1, Supplementary Table S1 previously reported in Palka *et al*.)^14^. A total of 3,158 sightings of cetacean species/groups were available for 1,413 of these spatial-temporal cells (273 for spring, 796 for summer, 257 for fall and 87 for winter). The species/groups sightings per season are summarized in Supplementary Table S2, (previously reported in Palka *et al*.)^14^ and the environmental covariates are summarized in Supplementary Table S3. Information related to effort and seasonal sightings were previously reported in Palka *et al*.^14^.

A total of 19 season/species predictive models were developed, which included 10 single species models using data from all seasons combined (spring, summer and fall), 6 species/group models using data from summer only, and 3 models for harbour porpoise which had sufficient sample size to develop separate seasonal models to explain fine scale seasonal migration for spring, summer and fall. Due to low effort and low animal density detected for most of the species, the winter data was not included in the model development, with the exception of the model for common bottlenose dolphin where sufficient numbers of animals were detected.

The most parsimonious model for each species included 4–6 different environmental covariates. The total deviance explained by the models ranged from 16.2% for the summer model of Atlantic spotted dolphin to 69.3% for the summer model of harbour porpoise. Overall, latitude and SST were included in 67% and 61% of the models respectively, followed by bottom temperature, primary productivity and sea floor slope in 39% of the models. The least frequent predictors included in the models were particulate organic carbon and sea surface height anomaly both with 5.6%. (Table 1).

**Table 1:** Percent deviance explained (DE) by term for each species model.

SPECIES	Generalized Additive Model Terms																	Total DE
	SST	BT	PP	CHL	PIC	POC	SAL	MLD	SLA	LAT	DEPTH	SLOPE	D2S	D125	D200	D1000	te(LAT, BT)	%
Atlantic spotted dolphin (summer)			3.59		2.52		3.43			6.66								16.2
Beaked whale, Cuvier’s (summer)	1.13	11.08								10.92	4.08	6.79						34.0
Beaked whale, Sowerby’s (summer)	6.49						1.09	3.54	22.96						7.02		41.1	
Beaked whale group (summer)	0.80									13.64	23.11	0.56						38.1
Common bottlenose dolphin (spring, summer, fall and winter)	0.4		3.11					1.02				0.19	1.48		2.88		13.22	22.3
Fin whale		7.86		0.98						15.79				10.06				34.7
Harbour porpoise (spring)			2.60							40.87			4.39		2.03			49.9
Harbour porpoise (summer)					1.48			4.70		60.17	2.95							69.3
Harbour porpoise (fall)	2.62									10.80			42.97		10.21			66.6
Humpback whale	9.14		6.48	0.65						9.97				5.66				31.9
Dwarf/Pygmy sperm whale group (summer)				1.41			0.57				28.27	0.80				2.65		33.7
Minke whale	5.88		9.59			4.51				10.70			5.65	3.57				39.9
Short/Long-finned pilot whale group		5.76	11.97		6.53					10.21		21.73						56.2
Risso’s dolphin	6.12	5.67										6.39	20.25	11.16				49.6
Sei whale	20.18							3.69					24.76	3.87				52.5
Common dolphin	14.53									9.39			10.53			7.64		42.1
Sperm whale	7.24	10.07	5.35	1.35								9.49						33.5
Striped dolphin (summer)	6.79	41.52	1.39													3.12		52.8
White-sided dolphin	4.38			2.63				2.90				1.02		7.57				18.5

The data input for the models cover spring, summer and fall seasons unless is specified in parenthesis. All the model terms were significant at p < 0.05 by a Wald-type test of f = 0^54^.

Habitat suitability maps generated from the model outputs identified clear differences in the core habitat for the species that have the tendency to converge in the same space and time, giving indications of habitat partition (Supplementary Figs S1 to S17).

Goodness-of-fit measures of the models were determined to be adequate as evaluated in two ways. First, overall the models fitted the input data well according to the Spearman’s rank correlation, mean absolute error, and mean absolute percent error statistics, using both the non-zero input data and cross-validation methods (Table 2). Second, a comparison of habitat suitability maps to field sighting locations revealed a good correspondence between the model predictions and observed data used to develop the models (Supplementary Figs S1 to S17).

**Table 2:** Results of diagnostic tests to evaluate fit of the habitat models.

Species	Non-Zero Input Data			k cross-validation k = 25		
	RHO	MAE	MAPE	RHO	MAE	MAPE
Atlantic spotted dolphin	0.273	0.199	87.861	0.113	0.195	86.271
Cuvier’s beaked whale	0.091	0.013	86.030	0.219	0.017	85.129
Sowerby’s beaked whale	0.379	0.003	92.233	0.151	0.004	93.303
Unidentified beaked whales	0.429	0.013	81.191	0.173	0.013	85.211
Common bottlenose dolphin	0.315	0.411	77.602	0.203	0.421	82.069
Fin whale	0.117	0.009	88.725	0.129	0.009	89.992
Harbour porpoise - Fall	0.179	0.038	85.778	0.189	0.047	78.802
Harbour porpoise - Spring	0.242	0.049	87.975	0.175	0.049	87.068
Harbour porpoise - Summer	0.260	0.113	80.405	0.209	0.102	79.875
Humpback whale	0.278	0.003	91.975	0.085	0.004	91.612
Pygmy/dwarf sperm whale	0.404	0.017	82.288	0.203	0.016	82.218
Minke whale	0.500	0.006	94.595	0.087	0.005	95.775
Long/short pilot whale	0.530	0.074	88.925	0.146	0.065	95.085
Risso’s dolphin	0.103	0.054	85.387	0.187	0.052	99.327
Sei whale	0.239	0.004	87.176	0.078	0.004	89.865
Common dolphin	0.192	0.277	104.383	0.183	0.324	105.963
Sperm whale	0.227	0.006	82.018	0.145	0.005	81.383
Striped dolphin	0.290	0.065	76.438	0.235	0.074	128.025
White-sided dolphin	0.314	0.303	86.471	0.064	0.265	92.461

RHO = Spearman’s rank correlation coefficient; MAE = Mean absolute error; MAPE = Mean absolute percentage error. Fit threshold values were taken from Kinlan *et al*.^49^ where: $$\begin{array}{llll}\mathrm{RHO}: & {\rm{Poor}}={\rm{x}} < 0.05 & {\rm{Fair}}\,{\rm{to}}\,{\rm{good}}=0.05 < ={\rm{x}} < 0.3 & {\rm{Excellent}}={\rm{x}} > 0.3\\ \mathrm{MAE}: & {\rm{Poor}}={\rm{x}} > 1 & {\rm{Fair}}\,{\rm{to}}\,{\rm{good}}=1 > ={\rm{x}} > 0.25\, & {\rm{Excellent}}={\rm{x}} < =0.25\\ \mathrm{MAPE}: & {\rm{Poor}}={\rm{x}} > 150 \%  & {\rm{Fair}}\,{\rm{to}}\,{\rm{good}}=150 \%  > ={\rm{x}} > 50 \%  & {\rm{Excellent}}={\rm{x}} < =50 \% .\end{array}$$

### Distribution-abundance connection with environmental covariates

**Figure 2:**
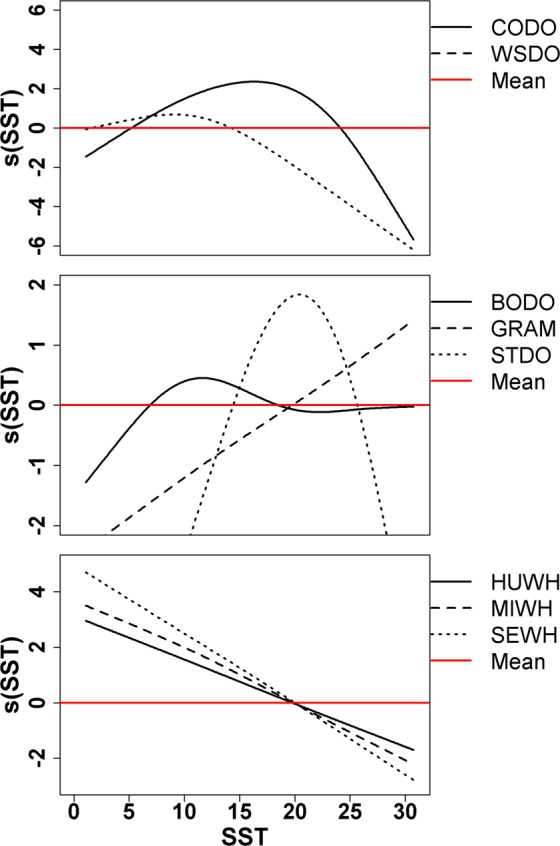
Examples of the partial effect of SST (°C) on the changes in abundance relative to its mean for common dolphin (CODO), white-sided dolphin (WSDO), common bottlenose dolphin (BODO), Risso’s dolphin (GRAM), striped dolphin (STDO), humpback whale (HUWH), minke whale (MIWH), sei whale (SEWH).

Partial regression smooth plots generated by the habitat models provided a good metric of the physical and biological habitat of these species, and represent how animal abundance changes relative to its mean in response to changes in each model covariate term. For example, the relationship between dolphin abundance and sea surface temperature showed well-defined thermal habitats that provide evidence of habitat partitioning (Fig. 2). This is illustrated by white-sided and bottlenose dolphins who are found more frequently in cooler waters, peaking at about 10 °C and 12 °C, respectively. Common and striped dolphins are found in warmer waters peaking at about 16 °C and 20 °C, respectively. In contrast, Risso’s dolphins are found most often in the warmest waters that are over 20 °C. The partial regression plots of sea surface temperature also provided information on the range of temperatures commonly used by the species. For example, common dolphins are found in waters with a wider range of temperatures (5–24 °C) in contrast to striped dolphins (14–25 °C).

**Figure 3:**
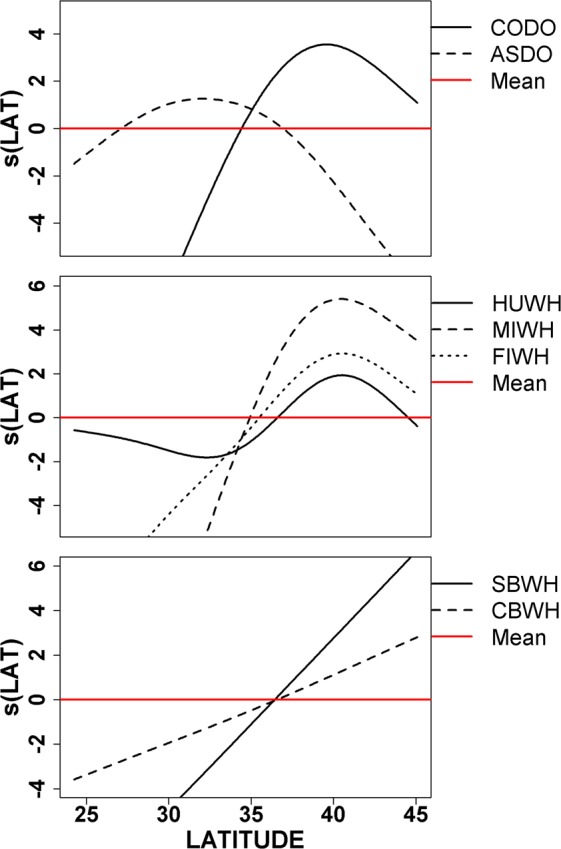
Examples of the partial effect of latitude on the changes in abundance relative to its mean for common dolphin (CODO), Atlantic spotted dolphin (ASDO), humpback whale (HUWH), minke whale (MIWH), fin whale (SEWH), Sowerby’s beaked whale (SBWH) and Cuvier’s beaked whale (CBWH).

The habitat covariates in the models were able to capture seasonal movement patterns and fine scale distribution patterns by including not only static covariates, like latitude, but also dynamic environmental covariates that change over the seasons and on a finer scale. For example, both humpback and minke whales have similar patterns in the partial regression relationships for sea surface temperature (Fig. 2) and latitude (Fig. 3). However, the humpback model also includes chlorophyll a and the minke whale model includes particulate organic carbon which aggregates phytoplankton, zooplankton, bacteria and detritus, suggesting a difference in trophic relationships and also resulting in very different habitat suitability patterns (Table 1, Supplementary Figs S8 and S10).

### Abundance estimates

Average seasonal abundance estimates derived from the habitat suitability models for the entire study area for all the cetacean species and species guilds are found in Table 3, these estimates were previously reported in Palka *et al*.^14^. For species that were detected during spring, summer and fall, various distribution and abundance patterns were observed. For the pilot whale, beaked whale, and *Kogia* groups, the abundance estimates were developed for the guild and not for individual species. In addition sightings of *Kogia* spp., beaked whales and striped dolphins occurred in habitats further offshore in regions only the shipboard surveys were able to access during summer. Consequently, the estimate of abundance for these species/groups only represents the summer season.

**Table 3:** Average seasonal abundance estimates derived from the 2010–2013 habitat models with its associated coefficient of variation in parenthesis.

Species	Spring (Mar- May)	Summer (Jun-Aug)	Fall (Sep-Nov)
Atlantic spotted dolphin	65,948 (0.16)	54,731 (0.15)	56,372 (0.16)
Beaked whale, Cuvier’s		3,425 (0.3)	
Beaked whale, Sowerby’s		676 (0.38)	
Beaked whale group		6,523 (0.17)	
Common bottlenose dolphin*	111,729 (0.38)	138,728 (0.37)	104,993 (0.24)
Fin whale	3,817 (0.15)	4,718 (0.13)	4,514 (0.12)
Harbour porpoise (spring)	30,126 (0.2)		
Harbour porpoise (summer)		83,250 (0.18)	
Harbour porpoise (fall)			17,943 (0.49)
Humpback whale	1,510 (0.23)	1,246 (0.17)	1,399 (0.17)
Dwarf/Pygmy sperm whale group		10,632 (0.18)	
Minke whale	1,484 (0.57)	2,834 (0.25)	2,829 (0.25)
Short/Long-finned pilot whale group	26,441 (0.4)	24,670 (0.3)	29,559 (0.3)
Risso’s dolphin	12,759 (0.21)	36,785 (0.2)	29,093 (0.21)
Sei whale	4,500 (0.42)	1,244 (0.47)	1,176 (0.48)
Common dolphin	111,042 (0.22)	118,697 (0.21)	183,510 (0.19)
Sperm whale	4,766 (0.33)	3,667 (0.14)	3,557 (0.15)
Striped dolphin		81,512 (0.12)	
White-sided dolphin	47,371 (0.49)	42,985 (0.46)	44,277 (0.39)

These estimates were previously reported in Palka *et al*.^14^.

### Robustness validation

**Figure 4:**
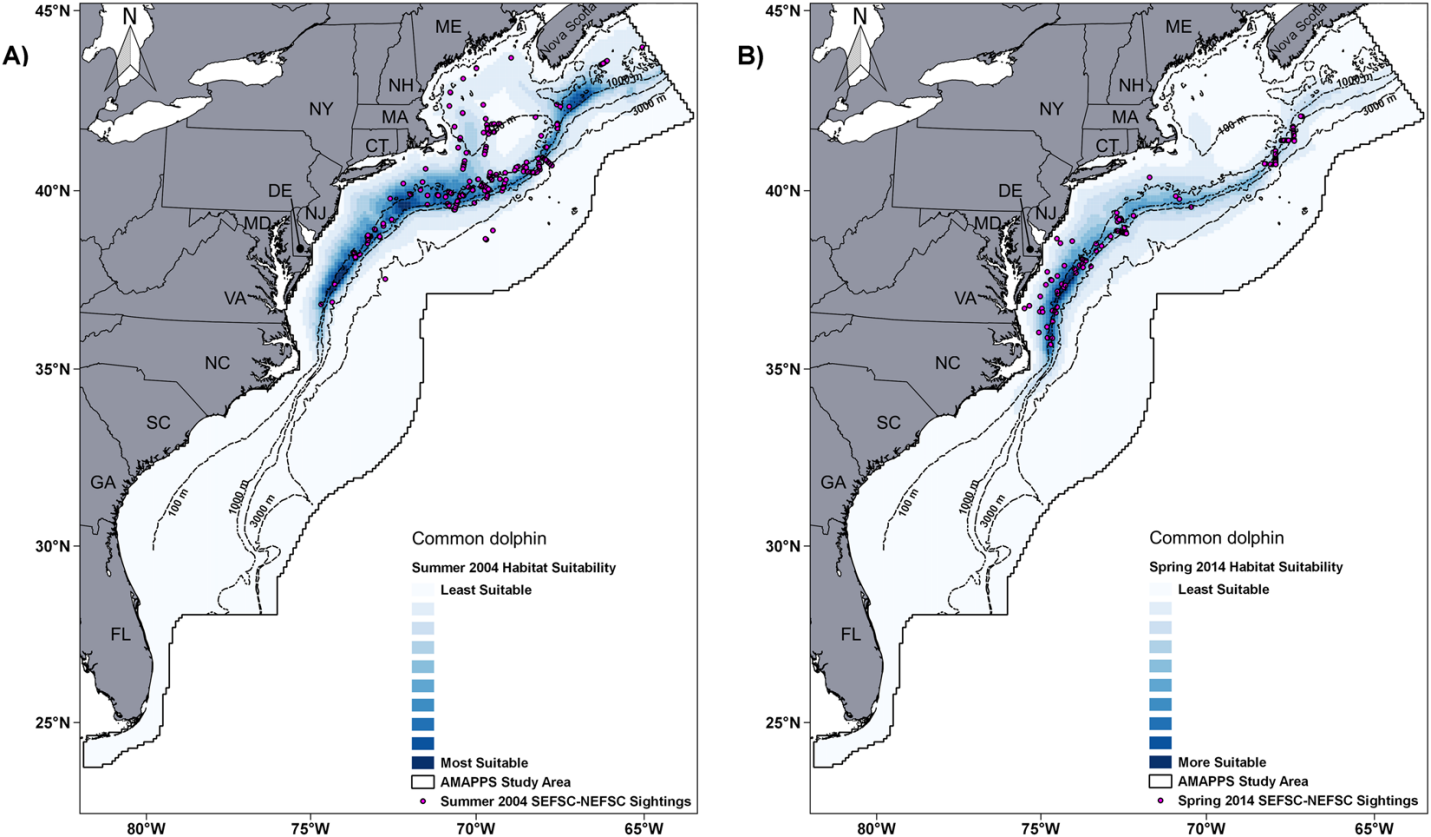
Robustness validation results of the common dolphin habitat model. Habitat suitability for (**A**) summer 2004; and (**B**) spring 2014 overlapped with the actual species sightings for the correspondent season and year. These sightings were not included in the habitat model development.

The models were also shown to be robust as defined by comparing the predicted model values to the data that were collected at times different than the data used to develop the models. Specifically, a total of 386 sightings were not included in the development of the models for humpback whale, fin whale, sperm whale, short/long-finned pilot whale, Risso’s dolphin, common dolphin and common bottlenose dolphin collected during spring 2014, were located within the core habitat regions as predicted by the habitat models when applied to the values of the covariates for the spring of 2014 (Supplementary Figs S18 to S24). In further analysis only for common dolphin, the 2010-2013 model definition applied to the 2004 summer environmental covariates predicted an abundance estimate that was less than 1% greater (not statistically different) than the previously reported 2004 abundance estimate when corrected for availability bias (Table 4). The previously reported 2004 estimate^15^ was produced from only data collected by shipboard and aerial surveys during summer of 2004. Even though the species distribution patterns detected during the surveys between summer 2004 and spring 2014 were quite different, the predicted habitat suitability maps matched the common dolphin sightings distribution recorded for both seasons and years (Fig. 4). Further indications of model fit are found in Palka *et al*.^14^ where the results from the goodness-of-fit tests of each the modelling steps are provided. In addition visual comparisons of the predicted seasonal density maps and locations of historical sightings since 1970 from OBIS-SEAMAP^16^ are also provided.

**Table 4:** Robustness validation results of the common dolphin model.

Source	Survey	Season	N_best_	ABC*	CN_best_	CV
Garrison *et al*.^55^	Shipboard	Jun-Aug	30,196	1.00	30,196	0.54
Palka *et al*.^56^	Shipboard	Jun-Aug	35,263	1.00	35,263	0.50
Palka *et al*.^56^	Aerial	Jun-Aug	55,284	0.93	59,445	0.24
SAR 2005^15^ (sum of the above)					124,904	0.23
Habitat model**					126,009	0.10

Common dolphin abundance estimates for 2004 by platform and the abundance estimate derived from the habitat model which includes the availability bias correction. N_best_ = Abundance estimate; ABC = Availability bias correction factor; CN_best_ = Abundance estimate corrected for availability bias; CV = Coefficient of variation. *Palka *et al*.^14^. **2010–2013 model definition, with June to August 2004 sea surface temperature data.

## Discussion

Federal agencies like U.S. Department of Energy, National Marine Fisheries Service, U.S. Fish and Wildlife Service, BOEM, and U.S. NAVY and other ocean developers require information from a diverse suite of topics such as density/abundance, distribution, stock structure, life history, behaviour, habitat use, environmental drivers, impact assessment and spatial modelling to support their mandates. Habitat models and model outputs presented in this paper provide some of the background information need by those agencies for spatial planning and conservation purposes and are available in a user friendly interface as a part of the AMAPPS model viewer at www.nefsc.noaa.gov/AMAPPSviewer. First, we will discuss the development of the models, and then compare these models to other developed in the same general region, then finally potential uses of the model results.

Partial regression smooth plots of the animal density in relation to SST and latitude generated by the models are in agreement with the current knowledge of the species distributions^17,18^. Despite model uncertainty, the robustness validation supported the predictive inferences of the models tested and expanded the potential application to detect species shifts in response to habitat changes. Though the amount of deviance explained is comparable to similar studies, improved deviance explained may be possible if additional physical and biological environmental covariates were included, such as fronts due to temperature, salinity and primary productivity, and densities of forage fish or other potential prey species.

One important assumption about regression type models, like those presented in this document, is that the models assume the link between animal density and habitat factors have consistent statistical relationships within the spatial-temporal variables included in the model. Given this assumption, it is then possible to predict the average density in locations or time periods where surveys did not actually occur^19^. However, if those proxies are unable to detect changes in the underlying ecological processes through time and space, then those assumptions are no longer valid. This means that a causal or mechanistic relationship is not explicitly assumed. Consequentially, the type of model used in this document provides an average pattern of the habitat suitability and abundance.

### Comparison with previous studies in the region

This effort is not the first of its kind for the western North Atlantic waters. The evolution of the theoretical and computational improvements related to modelling animal density is evident by the past and present efforts that have used line transect sightings data collected by the NEFSC and SEFSC. Hamazaki^17^ used multiple logistic regression to model the presence/absence of sightings from 1990–1996 with oceanographic and topographic variables to predict habitat maps. The U.S. Department of the Navy^20,21^ used sightings data from 1998–2005 in generalized additive density surface models to predict the density in a prediction grid, where g(0) was assumed to be one. The habitat suitability maps and abundance estimates presented in this document were generated with additional data that were not used in previous studies, and built upon past efforts. Most recently Roberts *et al*.^11^ used data from 1992–2014 to develop habitat-based climatological density maps and abundance estimates, where g(0) was not assumed to be one; though none of the data used in the current paper were used in the Roberts *et al*.^11^ models. In all of these efforts, the modelling approaches used the best available data and make logical assumptions and decisions.

Though making a direct comparison between these studies is a complex task, it is important to establish some of the fundamental differences between Roberts *et al*.^11^ and the present paper which include: (A) Differences in spatial and temporal coverage: most of the data used to develop the Roberts *et al*.^11^ models used data that were collected mostly from 1995 to 2009 (though there are some data from some species up to 2014) and did not include the data used in this paper, in addition the data were from surveys ranging from the US Pacific to Europe collected by multiple organizations and methods. In contrast, the models developed in this paper only use data from the area of interest. (B) Differences in analysis strategies and methods: to standardize all surveys used in Roberts *et al*.^11^ it was necessary to restrict data collected by only one sighting team per platform. Consequently, to correct for perception and availability bias, when local information was not available, it was necessary to apply correction factors from surveys conducted in the Pacific Ocean, Eastern Atlantic and Gulf of Mexico, which may result in unrepresentative corrections because of differences between the survey methods and animal’s behaviour. Finally, the current analyses also accounted for availability bias by estimating species-specific correction factors, some of which were estimated from recently tagged animals within the study area^14^. Another difference was the way the covariates were processed. For example, Robert’s developed climatological covariates which were a mean from 1995 to 2014 for a specific time period, say 8-days. In contrast, in the current paper the covariates were an average over a time period (8-days) from only the same year as the sighting observation. And (C) difference in presentation: average annual and monthly density-surface maps and abundances were presented in Roberts *et al*.^11^, in contrast to average seasonal estimates and the habitat suitability for the species presented in this study. In general, the average annual estimates from Roberts *et al*.^11^ are the most different from the seasonal estimates presented in this document for species that migrate out of US waters during some parts of the year, or for species that changed their seasonal spatial distribution patterns over the last two decades.

In summary, the two observer teams approach used in the present study that includes data from only the area of interest allowed the estimation of the perception bias correction from the same data that was used to calculate the density estimates thus resulting in regionally more representative and current abundance estimates and habitat suitability maps.

### Example applications of the habitat models

Society has increasing demands for energy production triggering the development of renewable energy areas on the outer continental shelf, currently reaching a total of 16,149 km^2^ from Massachusetts to Florida. These areas have a significant overlap with MMPA strategic dolphins and ESA whale species’ habitats and the level of interaction was documented and quantified by the models (Supplementary Table S4 and Figs S1 to S17). For example, even though most of the waters in the potential renewable energy areas are shallow and close to the shore, the models for pilot whales, Risso’s dolphins, white sided dolphins, common dolphins and Atlantic spotted dolphins identified the deeper offshore regions of the potential renewable energy areas as part of their preferred habitat. Interestingly, the model for sperm whales, which are generally considered deep water animals, predicted very low abundance at the farthest offshore regions of the potential renewable energy areas that were either close to the shelf break or extended into deeper waters like those in Massachusetts/Rhode Island and North Carolina and this has been confirmed by recorded sightings^14^.

There was a spatial difference in diversity patterns associated with latitude, in which the northern areas were more diverse in comparison to the southern areas. For example, the Massachusetts/Rhode Island area is located in a region with the highest species richness and showed the highest estimated abundance of ESA whale species (humpback, fin, sei and sperm whales) for all seasons. In addition the diversity index changed seasonally driven by animal migration (Supplementary Table S4). In the rest of the renewable areas the habitats become suitable for these species only during spring when the whales migrate through. In the case of MMPA strategic dolphins (harbour porpoises, white-sided dolphins, common dolphins, long/short finned pilot whales and common bottlenose dolphin), Massachusetts/Rhode Island, New Jersey and North Carolina/South Carolina showed the highest abundance estimates with similar spatial diversity patterns.

Changes in key physical and biological oceanographic features can alter marine ecosystems and atmospheric patterns. For example, in the Gulf of Maine spatial shifts of species assemblages associated with shallower, warmer waters tended to shift towards waters with cooler temperatures, while species assemblages associated with relatively cooler and deeper waters shifted deeper, but with little latitudinal change. Species assemblages associated with warmer and shallower water on the broad, shallow continental shelf from the Mid-Atlantic Bight to Georges Bank shifted strongly northeast along latitudinal gradients with little change in depth^22^. Habitat-based cetacean models such as those developed here will be able to be used to explore the potential changes in the distribution and abundance of cetaceans relative to the changes to the physical and biological changes.

It is clear that the effects on the movement and extent of species assemblages will hold important implications for management, mitigation and adaptation on these waters. The models and maps presented in this document provide a recent habitat characterization of the species discussed, and based on the assumptions and the predictive nature, have the potential to support management decisions and conservation measures in a marine spatial planning context.

## Methods

### Study area

The study area ranged from Halifax, Nova Scotia, Canada to the southern tip of Florida; from the coastline to slightly beyond the US exclusive economic zone and covers approximately 1,193,320 km^2^ (Fig. 1). It was subdivided into 10 × 10 km cells and sampled during 16 Atlantic Marine Assessment Program for Protected Species (AMAPPS) surveys, using NOAA Twin Otter aircrafts in coastal regions and NOAA ships *Henry B. Bigelow* by the Northeast Fisheries Science Center (NEFSC), and *Gordon Gunter* by the Southeast Fisheries Science Center (SEFSC) in offshore regions. These surveys covered approximately 103,995 km of line-transect survey effort during July 2010 to August 2013 (Supplementary Table S1). Habitat suitability models were built for 14 species and 3 species guilds (Table 1).

### Habitat predictors

Habitat predictors included a suite of static physiographic data and dynamic environmental covariates and were obtained from ETOPO1 1-min global relief data^23^, AVISO+^24^, the Hybrid Coordinate Ocean Model (HYCOM)^25^, and from NOAA’s Environmental Research Division Data Access Program (ERDDAP)^26^ website (Supplementary Table 3). The environmental data were downloaded from the source using a bounding box whose extent covered the study area, and subsequently processed using custom code developed in R (v. 3.1.1)^27^ with the R packages “raster” (v 2.5-2)^28^, “ncdf” (v 1.6.8)^29^, “rgdal” (v 1.1-6)^30^, “RNetCDF” (v 1.8-2)^31^, “lubridate” (v 1.5.3)^32^, “RODBC” (v 1.3–10)^33^ and “geosphere” (v 1.5–1)^34^. The process included a re-sampling of the data to the geographical midpoint of each 10 × 10 km stratum using oblique Mercator grid with bilinear interpolation. When possible, the data were obtained for dynamic covariates on an 8-day basis. Alternatively, daily images were downloaded and spatially synced to the cells and averaged into 8-day periods. In case of cells with missing values, a simple interpolation process was applied using the mean from the nearest-neighbour cells, and if needed the mean from the 8-day period before and after.

### Distance Analysis

Samples for modelling animal density were created by dividing the AMAPPS continuous survey effort into the 10 × 10 km cells. Species-specific information related to the number of sightings and group size was assigned to each cell. In addition, average sea state and glare within each cell was included as a continuous predictor variable to account for sighting conditions encountered on the surveyed track lines. Line-transect sightings parameter estimates derived from the surveys were based on effort in Beaufort Sea states from 0 through 4^35^, because the probability of detection decreases as the sea states increases^36,37^.

The density estimates were based on the independent observer approach assuming point independence^38^, calculated using the mark-recapture distance sampling (MRDS) with the computer program Distance (version 6.2)^39^, for each sampled 10 × 10 km cell using a Horvitz-Thompson-like estimator^40^. With this approach there was no need to independently model group size and the error due to extrapolation was minimized. To capture sightability differences between observation platforms and regions, data collected by each aircraft and ship from SEFSC and NEFSC surveys were analysed independently due to the differences in observers, data collection methods and habitats surveyed. A traditional MRDS distance analysis was used for the data collected by the shipboard surveys^35^. Data collected by the aerial surveys were analysed using a two-step process as described by Palka *et al*.^14^.

Significant covariates were chosen following the method suggested by Marques & Buckland^41^ and Laake & Borchers^38^. For all of the analyses, the detection probabilities were estimated using right truncated perpendicular distances as suggested in Buckland *et al*.^42^ and model selection was based on the goodness-of-fit using the AIC score (Akaike Information Criterion)^43^, Chi- squared test, Kolmogorov-Smirnov goodness-of-fit test, Cramer-von Mises goodness-of-fit test and a visual inspection of the fit, the results of these test are available in Palka *et al*.^14^. The estimated sighting probability included an estimation of g(0) which is the probability of detecting an animal on the survey track line.

To ensure sufficient sample sizes to accurately estimate model parameters, several similar species were pooled when needed. The criteria used to define species guilds included shape of the species’ detection functions, general animal behaviour, perceived sightability of the species, and sample size. The estimated global parameters were applied to the values of the covariates associated with each species in the species group to account for species-specific detection functions. An overall species-specific abundance estimate was then calculated for each cells/time period and corrected for species-specific availability bias by platform, as described in Palka *et al*.^14^. The availability bias correction was based on the probability of an animal being detectable at the surface during a survey, and took into consideration the species diving and aggregation behaviours, in addition to the amount of time the observer had to analyse any spot of water from each of the survey platforms. This correction tended to be larger for aerial surveys than for shipboard surveys, and larger for long diving species than for short diving species.

### Modelling

Generalized Additive Models (GAM)^44^ were developed in R (v. 3.1.1)^27^ using the package “mgcv” (v.1.8–6)^45^. The density estimates for each species/group in sampled cells by the shipboard and aerial surveys were defined as the response variable. The parameter estimates were optimized using restricted maximum likelihood criterion and the data were assumed to follow an overdispersed Tweedie distribution^46^ with null space penalization and thin plate splines with shrinkage^47^. Further, to avoid overfitting that could render parameter estimates difficult to interpret biologically, the maximum number of degrees of freedom was limited to 4. Correlations among environmental covariates ranged from 0.01–0.80 in absolute values. Although “mgcv” is considered to be robust to such correlations^45^, variables in a highly correlated pair above r = 0.60, were not used together in the same model.

Variable selection was performed with automatic term selection^48^ and a suite of diagnostic tests as proposed by Kinlan *et al*.^49^ and Barlow *et al*.^50^. Models with the lowest overall prediction errors and the highest percentage of deviance explained were selected for further diagnostic testing which included k-fold cross-validation with 25 random data subsets. K-fold cross-validation methods, in contrast to the method where data are partitioned into separate training and test sets, have the advantage of deriving a more accurate model, especially in cases with limited sample sizes^51^, such as in this study.

The relative importance of each term of the final model was estimated by calculating the terms’ approximate deviance explained following the process described by Whitlock *et al*.^52^. Briefly, this process involves fitting a sequence of models to obtain the deviance of the full model, null model and reduced models in which one smooth term was removed at a time, while retaining the other parameter estimates from the full model constant. Deviance explained (*DE*) for each term *i* was then calculated with the Eq. (1):

1 $$D{E}_{i}=(\frac{{D}_{ireducedmodel}-{D}_{Fullmodel}}{{D}_{nullmodel}})$$

where *D* is the deviance for a model, and *D*_*i reduced model*_ is a model where variable *i* is omitted.

Following model selection and validation for each of the species, the 2010–2013 average modelled seasonal (spring, summer and fall) abundance estimates for all cells in the study area were used to generate habitat suitability maps using QGIS (v. 2.10)^53^.

The habitat suitability (HS) was assumed to be directly correlated with the species’ abundance and distribution. That is, in times and regions with the greatest estimated abundance it was assumed that the habitat was the most suitable for the species, then using the Eq. (2):

2 $$HS=\sum _{i}^{n}\,\widehat{{N}_{i}}$$

where $\widehat{{N}_{i}}$ was the seasonal estimated abundance for each cell from the species-specific model. The seasonal abundance estimates were calculated by summing the mean predicted abundance of each cell, and the uncertainty estimates reflect only the uncertainty in the GAM parameter estimates.

Abundance estimates for smaller scale regions within study area that are being considered for development of offshore renewable energy were also summarized. In some cases it was needed to merge several wind energy lease areas/wind planning areas together when the areas were relatively small and close together. In addition a buffer zone was added around all areas in an attempt to designate a generic area in which an animal may be exposed to due to construction/operation activities within the renewable energy area. The size of an appropriate buffer is dependent on a variety of factors including species-specific factors, such as natural short-term foraging and movement patterns which could then influence the animal’s response and sensitivity to the activity. In addition, the types of activities being undertaken in the offshore renewable energy area, and the physical topography and oceanographic features have a direct impact on the sound level and propagation. However, for simplicity in this study, offshore wind energy areas in addition to 10 km buffer zone is referred to as renewable energy areas and is reflected in the abundance estimates.

Robustness validation of the habitat models was investigated in two ways. First the 2010–2013 model parameters were applied to the spring 2014 environmental data, the resulting predicted habitat suitability was compared with the actual spring 2014 sightings locations from the AMAPPS surveys. From the species included in this document, only common dolphin, Risso’s dolphin, common bottlenose dolphin, short/long-finned pilot whale, fin whale, humpback whale and sperm whale were detected during the surveys, thus only the habitat models of these species were included in the comparison. The second way was by hindcasting the 2010–2013 models by using the summer 2004 environmental data. But given the quality of the environmental data needed for the models were not readily available for the entire study area for 2004, the test was restricted to the common dolphin model. Thus, the modelled output was compared to not only the summer 2004 sightings locations from a NEFSC abundance survey but also the abundance estimate reported in the 2005 Stock Assessment Report^15^ that was derived from the NEFSC 2004 summer abundance survey data. The work presented in this document conforms to accepted international ethical standards.

## Supplementary information

Supplementary Information

## Data Availability

The datasets generated during the current study are available at https://inport.nmfs.noaa.gov/inport/item/23306 under “Distribution information”.

## References

[1] Ecosystem Assessment Program. Ecosystem Assessment Report for the Northeast U.S. Continental Shelf Large Marine Ecosystem. US Dept Commer, Northeast Fish Sci Cent Ref Doc. 09–11. Available at, https://www.nefsc.noaa.gov/publications/crd/crd0911/crd0911.pdf (2009).

[2] Perrin, W. F., Würsig, B. & Thewissen, J. G. M. (eds). *Encyclopedia of marine mammals*. 2nd ed. (Academic Press, 2009).

[3] Kanaji Y, Okazaki M, Kishiro T, Miyashita T (2015). Estimation of habitat suitability for the southern form of the short-finned pilot whale (*Globicephala macrorhynchus*) in the North Pacific. Fish. Oceanogr..

[4] Bowen WD (1997). Role of marine mammals in aquatic ecosystems. Marine Ecology Progress Series.

[5] Estes JA, Tinker MT, Willians TM, Doak DF (1998). Killer Whale Predation on Sea Otters Linking Oceanic and Nearshore Ecosystems. Science.

[6] Saba VS (2016). Enhanced warming of the Northwest Atlantic Ocean under climate change. J. Geophys. Res. Oceans.

[7] U.S. Department of Energy & U.S. Department of Interior. National Offshore Wind Strategy. DEO/GO-102016-4866 Available at, https://www.energy.gov/sites/prod/files/2016/09/f33/National-Offshore-Wind-Strategy-report-09082016.pdf (2016).

[8] Guisan A, Thuiller W (2005). Predicting species distribution: offering more than simple habitat models. Ecology Letters.

[9] Elith J, Leathwick JR (2009). Species distribution models: ecological explanation and prediction across space and time. Annual Review of Ecology and Evolutionary Systematics.

[10] Forney K, Becker E, Foley D, Barlow J, Oleson E (2015). Habitat-based models of cetacean density and distribution in the central North Pacific. Endang Species Res.

[11] Roberts JJ (2016). Habitat-based cetacean density models for the US Atlantic and Gulf of Mexico. Scientific Reports.

[12] Baltensperger AP, Huettmann F (2015). Predicted Shifts in Small Mammal Distributions and Biodiversity in the Altered Future Environment of Alaska: An Open Access Data and Machine Learning Perspective. PLoS ONE.

[13] Department of Commerce. Taking and Importing Marine Mammals; Taking Marine Mammals Incidental to the U.S. Navy Training and Testing Activities in the Atlantic Fleet Training and Testing Study Area. Federal Register Vol. 82, No. 155: 37851 (2017).

[14] Palka, D. L. *et al*. Atlantic Marine Assessment Program for Protected Species: 2010–2014 US Dept. of the Interior, Bureau of Ocean Energy Management, Atlantic OCS Region, Washington, DC. OCS Study BOEM 2017-071. Available at, https://www.boem.gov/espis/5/5638.pdf (2017).

[15] Waring, G. T., Josephson, E., Fairfield, C. P., & Maze-Foley K. (eds). U.S. Atlantic and Gulf of Mexico Marine Mammal Stock Assessments – 2005 NOAA Tech Memo 194 Available at, https://www.nefsc.noaa.gov/publications/tm/tm194/ (2006).

[16] Halpin PN (2009). OBIS-SEAMAP: The Word Data Center for Marine Mammals, Sea Birds, and Sea Turtle Distributions. Oceanography.

[17] Hamazaki T (2002). Spatiotemporal prediction models of cetacean habitats in the mid‐Western North Atlantic Ocean (from Cape Hatteras, North Carolina, USA to Nova Scotia, Canada). Marine Mammal Science.

[18] Selzer LA, Payne PM (1988). The distribution of white-sided (Lagenorhynchus acutus) and common dolphins (Delphinus delphis) vs. environmental features of the continental shelf of the Northeastern United States. Marine Mammal Science.

[19] Guisan A, Edwards JTC, Hastie T (2002). Generalized linear and generalized additive models in studies of species distributions: setting the scene. Ecological Modelling.

[20] Department of the Navy. Navy OPAREA Density Estimates (NODE) for the Southeast OPAREAS: VACAPES, CHPT, JAX/CHASN, and Southeastern Florida & AUTEC-Andros Available at, http://seamap.env.duke.edu/downloads/resources/serdp/Northeast%20NODE%20Final%20Report.pdf (2007).

[21] Department of the Navy. Navy OPAREA Density Estimates (NODE) for the Northeast OPAREAS: Boston, Narragansett Bay and Atlantic City Available at, http://seamap.env.duke.edu/downloads/resources/serdp/Southeast%20NODE%20Final%20Report.pdf (2007).

[22] Kleisner KM (2016). The Effects of Sub-Regional Climate Velocity on the Distribution and Spatial Extent of Marine Species Assemblages. PLoS ONE.

[23] Amante, C. & Eakins, B. W. ETOPO1 1 Arc-Minute Global Relief Model: Procedures, Data Sources and Analysis. NOAA Technical Memorandum NESDIS NGDC-24. National Geophysical Data Center, NOAA. 10.7289/V5C8276M [access date: 11/17/14] (2009).

[24] AVISO+. The Ssalto/Duacs altimeter products were produced and distributed by the Copernicus Marine and Environment Monitoring Service (CMEMS), http://www.marine.copernicus.eu [access date: 11/20/14].

[25] Chassignet EP (2007). The HYCOM (Hybrid Coordinate Ocean Model) data assimilative system. J. Mar. Syst..

[26] Simons, R. A. ERDDAP, http://coastwatch.pfeg.noaa.gov/erddap. Monterey, CA: NOAA/NMFS/SWFSC/ERD [access date: 11/17/14] (2015).

[27] R Core Team. R: A language and environment for statistical computing. R Foundation for Statistical Computing, Vienna, Austria, http://www.R-project.org/ (2014).

[28] Hijmans, R. J. & van Etten J. Raster: Geographic analysis and modeling with raster data. R package version 2.0–12, http://CRAN.R-project.org/package=raster (2012).

[29] Pierce, D. ncdf: Interface to Unidata netCDF data files. R package version 1.6.8, http://CRAN.R-project.org/package=ncdf (2014).

[30] Bivand, R., Keitt, T. & Rowlingson, B. rgdal: Bindings for the Geospatial Data Abstraction Library. R package version 1.0–4, http://CRAN.R-project.org/package=rgdal (2015).

[31] Michna, P. & Woods, M. RNetCDF: Interface to NetCDF Datasets. R package version 1.8–2, http://CRAN.R-project.org/package=RNetCDF (2016).

[32] Grolemund, G. & Wickham, H. Dates and Times Made Easy with lubridate. *Journal of Statistical Software* 40, 1–25, http://www.jstatsoft.org/v40/i03/ (2011).

[33] Ripley, B. & Lapsley, M. RODBC: ODBC Database Access. R package version 1.3–10, https://CRAN.R-project.org/package=RODBC (2015).

[34] Hijmans, R. J. geosphere: Spherical Trigonometry. R package version 1.5–1, https://CRAN.R-project.org/package=geosphere (2015).

[35] Palka, D. Cetacean abundance estimates in US northwestern Atlantic Ocean waters from summer 2011 line transect survey. US Dept Commer, Northeast Fish Sci Cent Ref Doc. 12–29; 37 p. Available from: National Marine Fisheries Service, 166 Water Street, Woods Hole, MA 02543-1026, or online at, http://www.nefsc.noaa.gov/nefsc/publications/ (2012).

[36] Palka D (1996). Effects of Beaufort Sea State on the Sightability of Harbor Porpoises in the Gulf of Maine. REP. INT. WHAL. COMMN.

[37] Barlow J, Gerrodette T, Forcada J (2001). Factors affecting perpendicular sighting distances on shipboard line-transect surveys for cetaceans. Journal of Cetacean Research and Management.

[38] Laake, J. & Borchers, D. In *Advanced distance sampling* (Buckland, S. T., Anderson, D. R., Burnham, K. P., Laake, J. L. & Thomas, L. eds) 108–189 Oxford University Press (2004).

[39] Thomas L (2010). Distance software: design and analysis of distance sampling surveys for estimating population size. Journal of Applied Ecology.

[40] Borchers DL, Laake JL, Southwell C, Paxton CGM (2006). Accommodating Unmodeled Heterogeneity in Double-Observer Distance Sampling Surveys. Biometrics.

[41] Marques FFC, Buckland ST (2003). Incorporating covariates into standard line transect analyses. Biometrics.

[42] Buckland, S. T. *et al*. *Introduction to distance sampling: estimating abundance of biological populations*. (Oxford University Press, 2001).

[43] Akaike H (1974). New look at statistical-model identification. IEEE Transactions on Automatic Control AC.

[44] Hastie, T. J. & Tibshirani, R. J. Generalized additive models. (Chapman & Hall/CRC, 1990). 10.1177/096228029500400302 8548102

[45] Wood SN (2011). Fast stable restricted maximum likelihood and marginal likelihood estimation of semiparametric Generalized Linear Models. J. R. Stat. Soc. Ser. B.

[46] Miller DL, Burt ML, Rexstad EA, Thomas L, Gimenez O (2013). Spatial models for distance sampling data: Recent developments and future directions. Methods Ecol. Evol..

[47] Wood SN, Augustin NH (2002). GAMs with integrated model selection using penalized regression splines and applications to environmental modeling. Ecol. Model..

[48] Marra G, Wood S (2011). Practical variable selection for generalized additive models. Comput. Stat. Data Anal..

[49] Kinlan, B. P., Menza,C. & Huettmann, F. In *A biogeographic assessment of seabirds, deep sea corals and ocean habitats of the New York bight: Science to support offshore spatial planning* (Menza, C., Kinlan, B. P., Dorfman, D. S., Poti, M. & Caldow, C. eds) 87–148 (NOAA Technical Memorandum NOS NCCOS 141 2012).

[50] Barlow, J. *et al*. Predictive modeling of cetacean densities in the eastern Pacific Ocean. U.S. Department of Commerce, NOAA Technical Memorandum, NMFS-SWFSC-444. 206p. Available at, https://swfsc.noaa.gov/publications/TM/SWFSC/NOAA-TM-NMFS-SWFSC-444.pdf (2009).

[51] Seni G, Elder F (2010). Ensemble Methods in Data Mining: Improving Accuracy Through Combining Predictions. Synthesis Lectures on. Data Mining and Knowledge Discovery.

[52] Whitlock RE (2015). Direct quantification of energy intake in an apex marine predator suggests physiology is a key driver of migrations. Sci. Adv..

[53] QGIS Development Team. QGIS Geographic Information System. Open Source Geospatial Foundation Project, http://qgis.osgeo.org (2009).

[54] Wood SN (2013). On p-values for smooth components of an extended generalized additive model. Biometrika.

[55] Garrison LP, Martinez A, Maze-Foley K (2010). Habitat and abundance of cetaceans in Atlantic Ocean continental slope waters off the eastern USA. Journal of Cetacean Research and Management.

[56] Palka, D. Summer abundance estimates of cetaceans in US North Atlantic Navy Operating Areas U.S. Dep. Commer., Northeast Fish. Sci. Cent. Ref. Doc. 06-03; 41 p. Available at, https://www.nefsc.noaa.gov/publications/crd/crd0603/crd0603.pdf (2006).

