# Supplementary material for: Environmental predictors of habitat suitability and occurrence of cetaceans in the western North Atlantic Ocean
Source: Sci Rep. 2019 Apr 9;9:5833. doi: 10.1038/s41598-019-42288-6 (PMC6456503; doi:10.1038/s41598-019-42288-6)
Supplement: Supplementary file 1 — Supplementary Information [file 41598_2019_42288_MOESM1_ESM.pdf]

Environmental predictors of habitat suitability and occurrence of cetaceans in the western North Atlantic Ocean.

Supplementary Information

Samuel Chavez-Rosales, Debra L. Palka, Lance P. Garrison & Elizabeth A. Josephson.

Supplementary Tables

Supplementary Table S1. Summary of effort per season and platform. These data were previously reported in Palka *et al.*[1].

Platform	Effort (km)				
	Spring	Summer	Fall	Winter	Total
NE Shipboard	0	8,146	0	0	8,146
NE Aerial	7,502	10,468	11,038	3,573	32,581
SE Shipboard	0	8,537	2,093	0	10,630
SE Aerial	17,978	16,835	11,818	6,007	52,638
Total	25,480	43,986	24,949	9,580	103,995

Supplementary Table S2. Summary of marine mammal sightings per season. These data were previously reported in Palka *et al.*[1].

Specie	Total sightings / Total Animals			
	**Spring**	**Summer**	**Fall**	**Winter**
Atlantic spotted dolphin	32 / 481	145 / 4,575	40 / 926	7 / 385
Beaked whale, Cuvier's	2 / 2	103 / 248	5 / 9	0 / 0
Beaked whale, Sowerby's	0 / 0	27 / 75	0 / 0	0 / 0
Beaked whale group	4 / 8	110 / 279	5 / 11	0 / 0
Common bottlenose dolphin	253 / 2222	515 / 6974	210 / 2940	82 / 542
Fin whale	31 / 45	117 / 157	34 / 45	4 / 4
Harbour porpoise	129 / 181	351 / 1,238	50 / 128	68 / 92
Humpback whale	19 / 23	86 / 119	31 / 45	4 / 4
Dwarf/Pygmy sperm whale group	0 / 0	115 / 201	2 / 2	0 / 0
Minke whale	12 / 13	52 / 52	23 / 34	1 / 1
Short/Long-finned pilot whale group	4 / 139	199 / 2,810	59 / 780	5 / 6
Risso's dolphin	33 / 139	257 / 1,646	24 / 192	23 / 61
Sei whale	5 / 6	11 / 12	3 / 9	3 / 6
Common dolphin	73 / 3,278	264 / 9,418	67/1,525	19 / 633
Sperm whale	9 / 9	195 / 372	17 / 46	0 / 0
Striped dolphin	2 / 210	139 / 6,101	7 / 325	0 / 0
White-sided dolphin	37 / 366	26 / 442	13 / 315	18 / 132

Supplementary Table S3. Environmental covariates included in the habitat models.

Abbreviation	Resolution	Description	Source
DEPTH	3 arcsec	Bathymetry (m)	ETOPO1[2]
D2S	0.04º	Distance to coastline (m)	https://oceancolor.gsfc.nasa.gov
SLOPE	3 arcsec	Seafloor slope (º)	ETOPO1[2]
D200	1’	Distance to 200 m isobath	ETOPO1[2]
D125	1’	Distance to 125 m isobath	ETOPO1[2]
D1000	1’	Distance to 1000 m isobath	ETOPO1[2]
SST	0.05º	Sea surface temperature (°C)	ERDDAP[3]
LAT		Latitude (º)	
CHL	0.0125º /0.04166º	Chlorophyll a (mg m-3)	ERDDAP[3]
PP	0.1º	Primary productivity (mgC m−2 yr−1)	ERDDAP[3]
PIC	4 km	Particulate inorganic carbon (mol m-3)	ERDDAP[3]
POC	4 km	Particulate organic carbon (mg m-3)	ERDDAP[3]
BT	1/12º	Bottom temperature (°C)	HYCOM[4]
SAL	1/12º	Salinity (psu)	HYCOM[4]
SLA	1/4º	Sea surface height anomaly	AVISO+[5]
MLD	1/12º	Mix layer depth, depth at which the density changes from the surface by 0.03 kg/m3 (m)	HYCOM[4]

Supplementary Table S4. Seasonal estimates of species richness (SR), Shannon diversity index (H’) and average abundance for ESA whale species and MMPA strategic dolphins in the renewable energy areas.

Renewable Energy Areas				ESA whale species		MMPA Strategic dolphins	
Season	Location	SR	H’	Abundance	CV	Abundance	CV
Spring	Massachusetts/ Rhode Island	9	1.34	96	0.070	3,147	0.058
	New York	9	1.39	13	0.087	593	0.069
	New Jersey	9	0.97	27	0.080	1,801	0.091
	Delaware/Maryland	9	0.94	19	0.071	1,443	0.073
	Virginia	8	1.03	13	0.080	1,263	0.062
	North Carolina	8	1.01	24	0.092	3,552	0.057
	North Carolina/South Carolina	6	0.20	7	0.207	7,517	0.089
	Georgia	2	0.01	1	0.274	988	0.107
	Florida	3	0.42	2	0.104	117	0.229
Summer	Massachusetts/ Rhode Island	9	0.91	87	0.060	4,526	0.057
	New York	8	0.73	6	0.096	1042	0.143
	New Jersey	8	0.29	4	0.142	6,587	0.287
	Delaware/Maryland	7	0.41	2	0.131	2,746	0.127
	Virginia	7	0.84	2	0.144	625	0.078
	North Carolina	8	1.08	5	0.092	1,281	0.070
	North Carolina/South Carolina	4	0.02	0		11,310	0.076
	Georgia	1	0	0		1,857	0.089
	Florida	2	0.24	0		168	0.275
Fall	Massachusetts/ Rhode Island	9	0.83	89	0.058	4,739	0.054
	New York	9	0.92	9	0.095	921	0.100
	New Jersey	9	0.72	15	0.105	3,408	0.102
	Delaware/Maryland	9	0.76	9	0.107	2,284	0.077
	Virginia	8	0.79	4	0.107	1,234	0.062
	North Carolina	8	0.75	8	0.089	2,916	0.059
	North Carolina/South Carolina	4	0.05	0		8,328	0.083
	Georgia	1	0	0		1,316	0.098
	Florida	2	0.34	0		127	0.272

Supplementary Figures

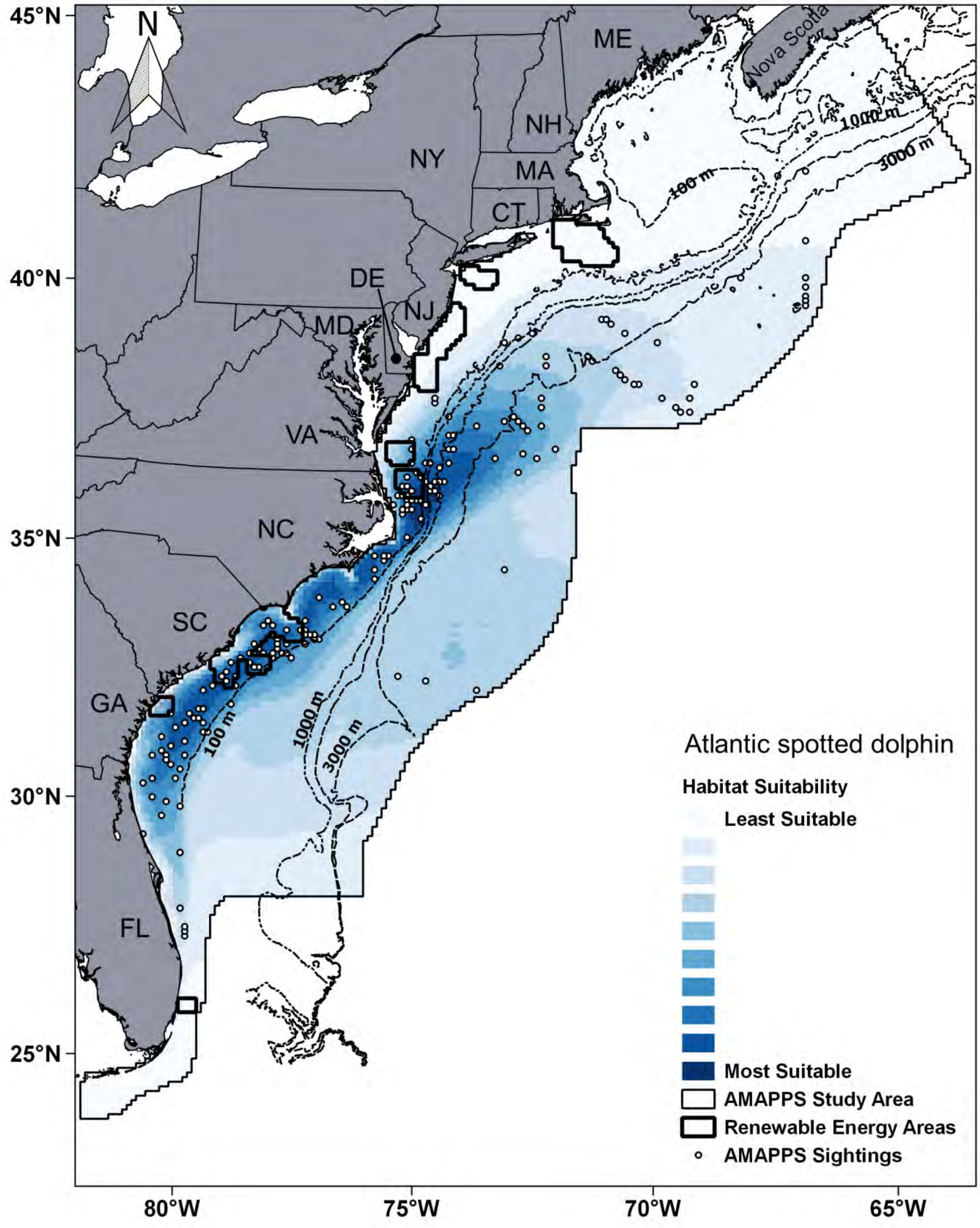

Supplementary Figure S1. Summer habitat suitability for Atlantic spotted dolphin (*Stenella frontalis*). White circles indicate cells with one or more animal sightings. Renewable energy areas include a 10 km buffer zone.

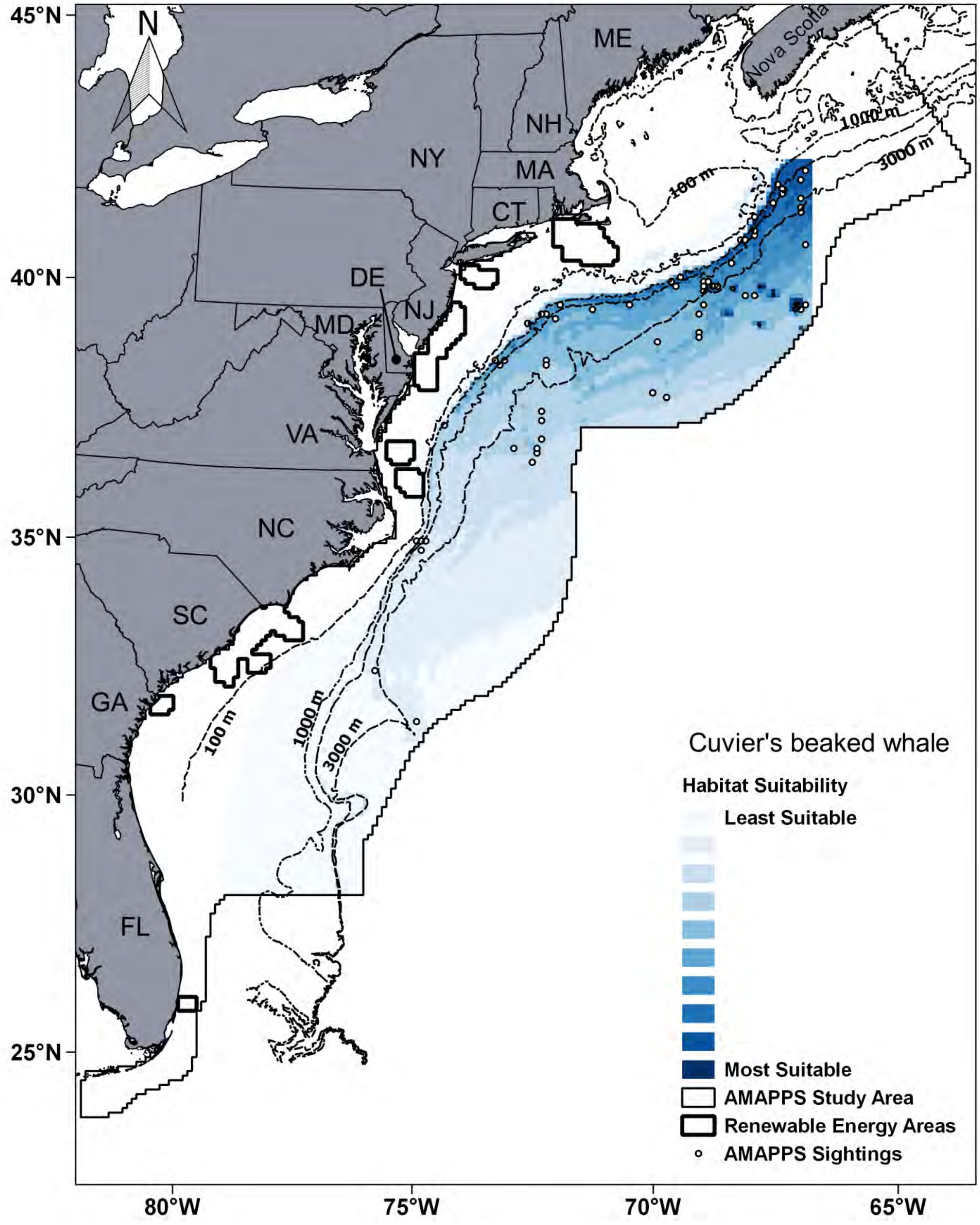

Supplementary Figure S2. Summer habitat suitability for Cuvier's beaked whale (*Ziphius cavirostris*). White circles indicate cells with one or more animal sightings. Renewable energy areas include a 10 km buffer zone.

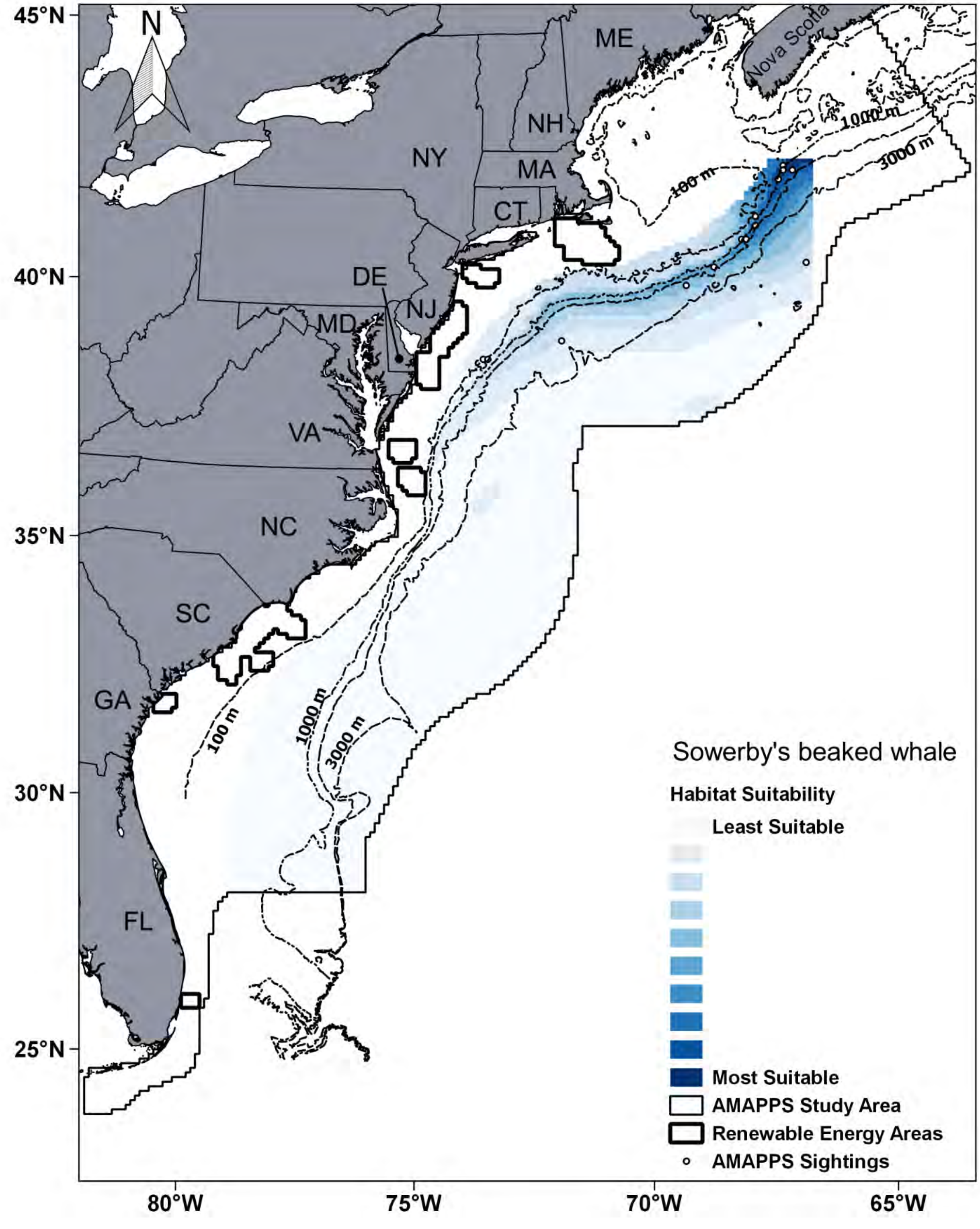

Supplementary Figure S3. Summer habitat suitability for Sowerby's beaked whale (*Mesoplodon bidens*). White circles indicate cells with one or more animal sightings. Renewable energy areas include a 10 km buffer zone.

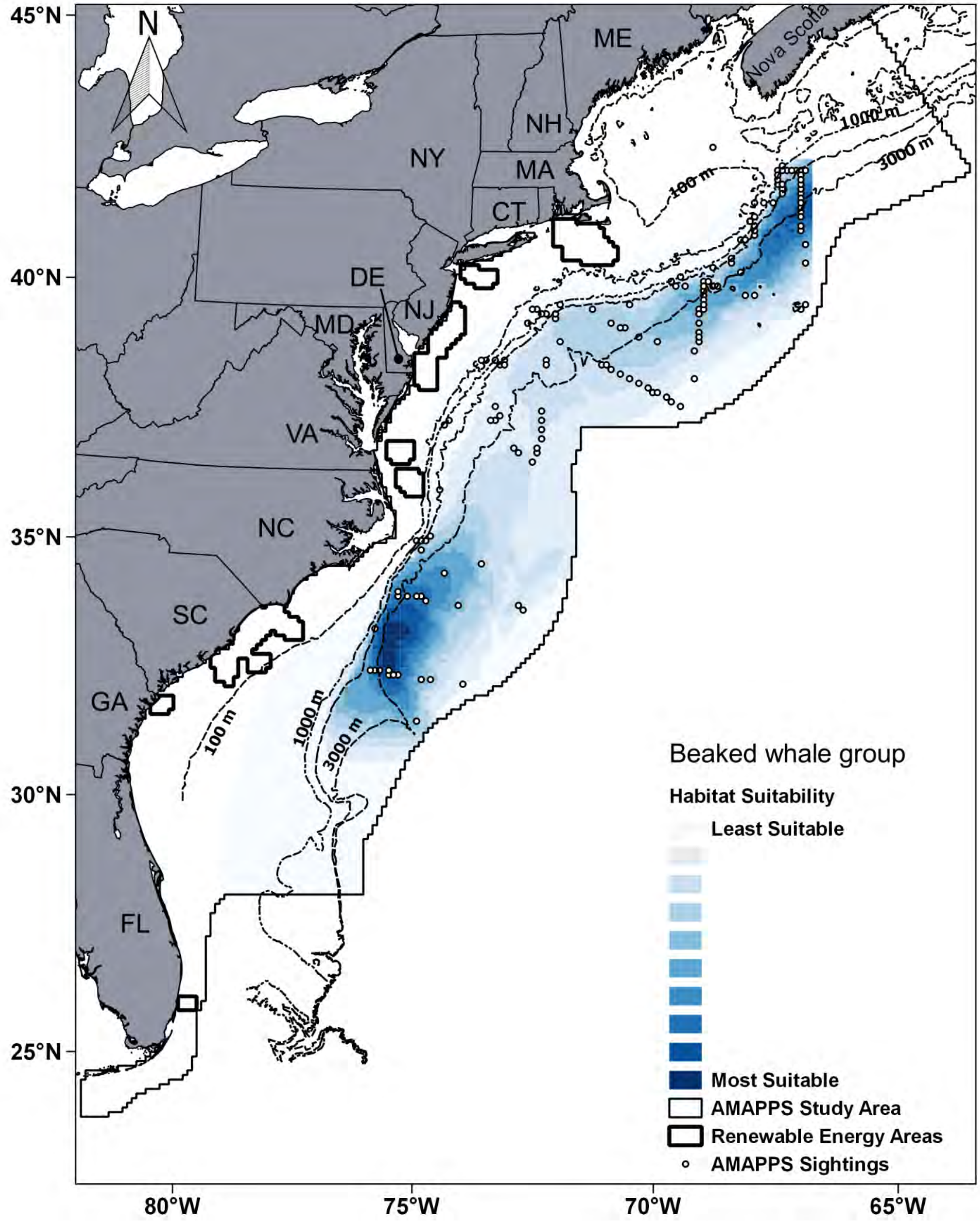

Supplementary Figure S4. Summer habitat suitability for beaked whale species. White circles indicate cells with one or more animal sightings. Renewable energy areas include a 10 km buffer zone.

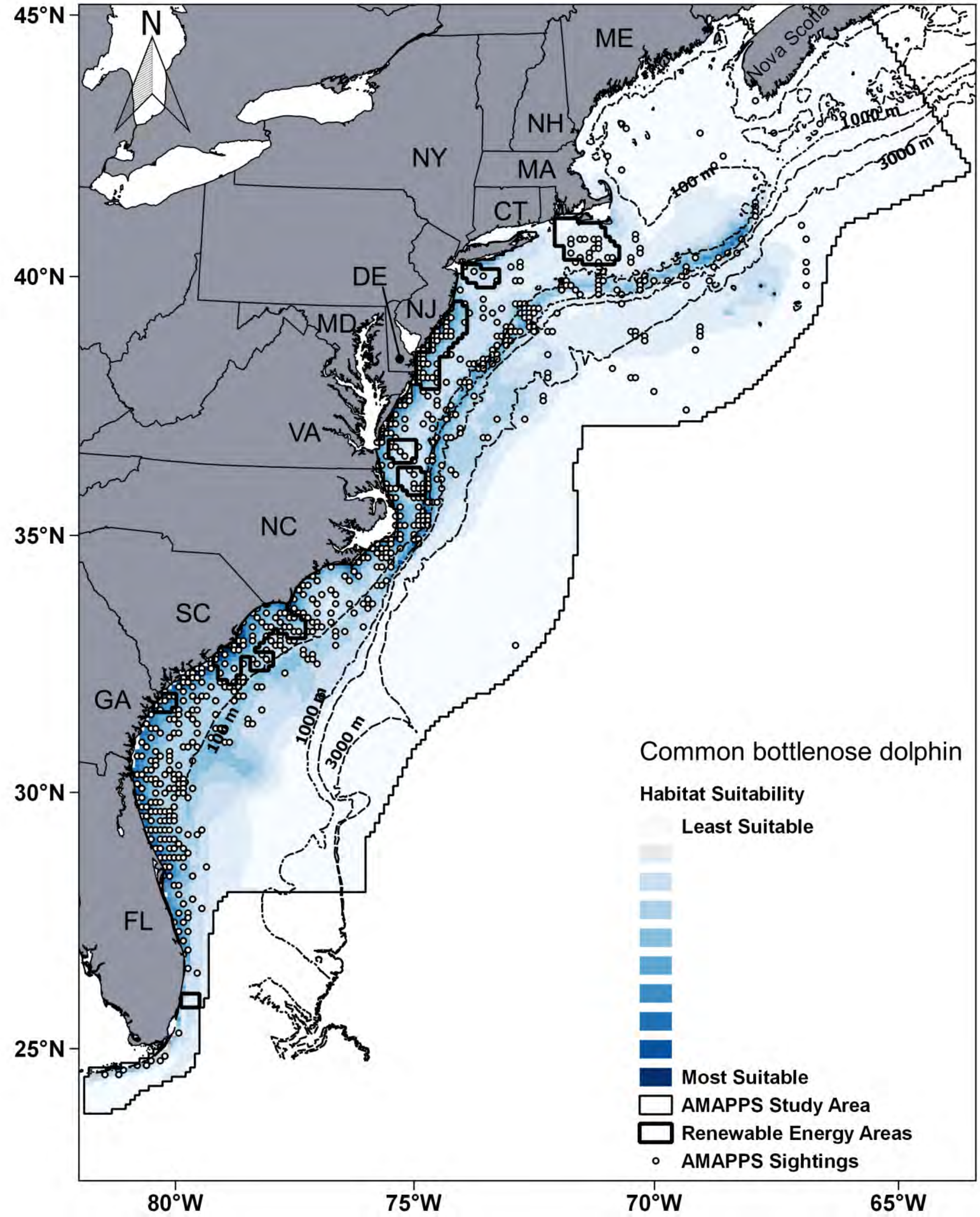

Supplementary Figure S5. Habitat suitability for common bottlenose dolphin (*Tursiops truncatus*). White circles indicate cells with one or more animal sightings. Renewable energy areas include a 10 km buffer zone.

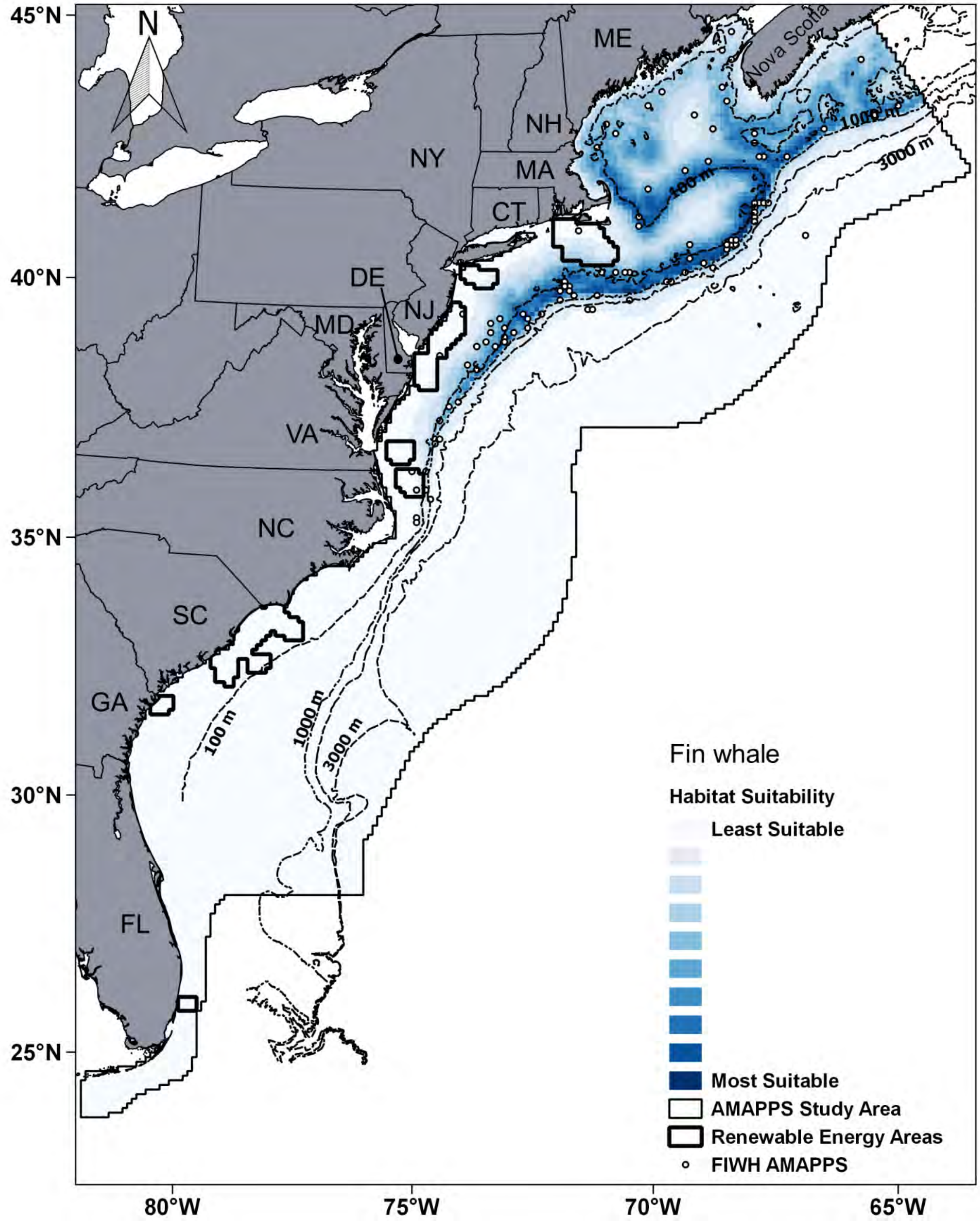

Supplementary Figure S6. Habitat suitability for fin whale (*Balaenoptera physalus*). White circles indicate cells with one or more animal sightings. Renewable energy areas include a 10 km buffer zone.

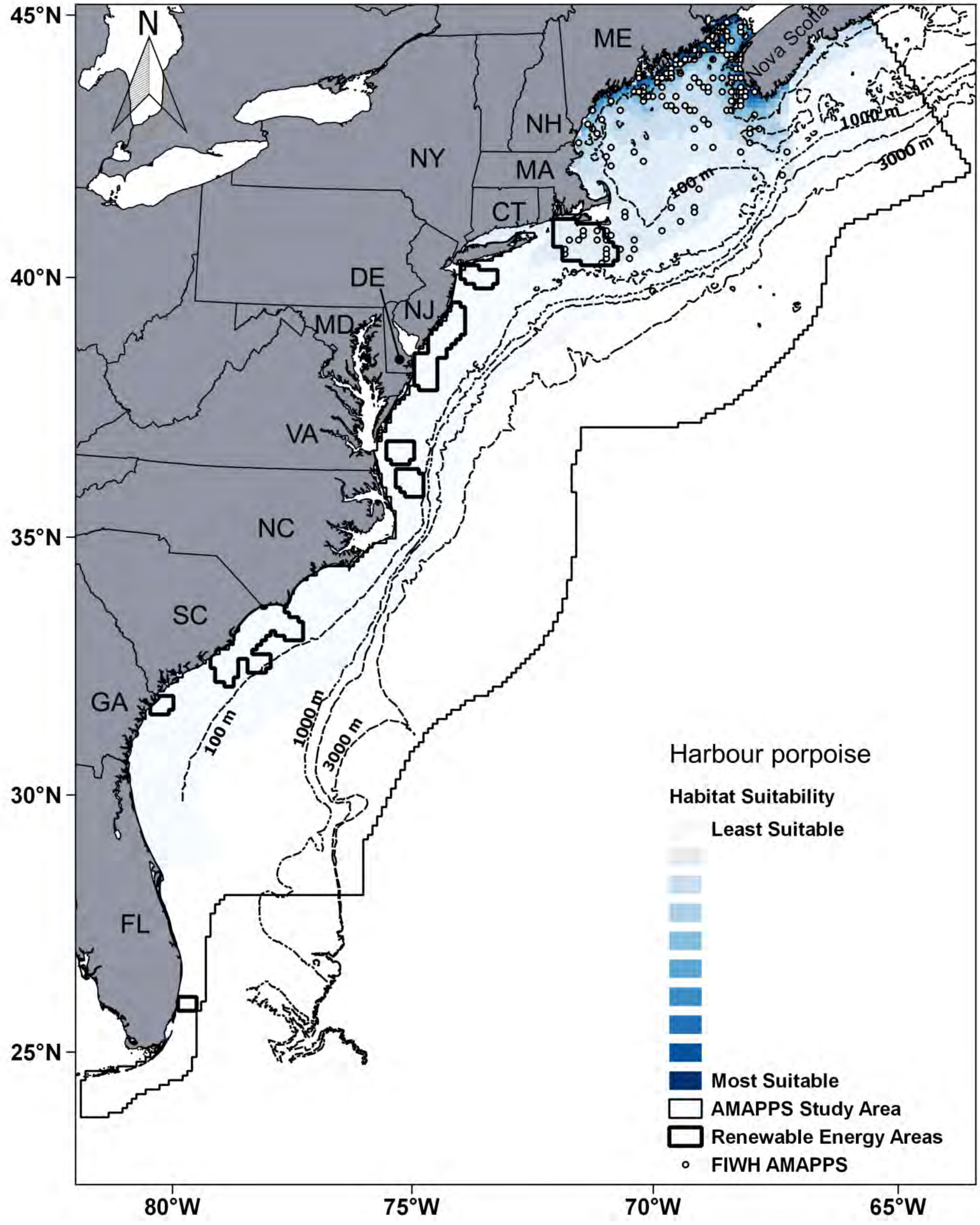

Supplementary Figure S7. Habitat suitability for harbour porpoise (*Phocoena phocoena*). White circles indicate cells with one or more animal sightings. Renewable energy areas include a 10 km buffer zone.

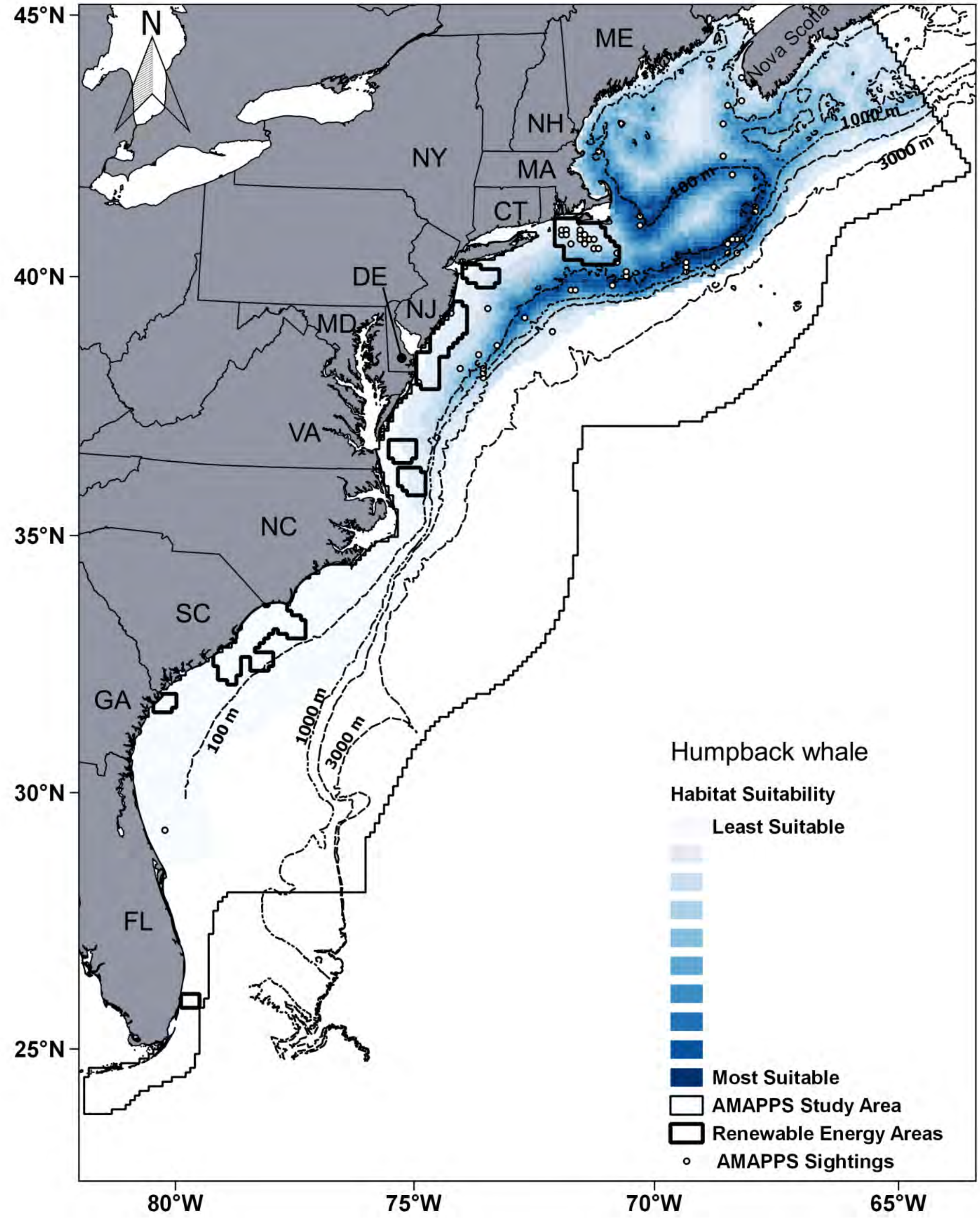

Supplementary Figure S8. Habitat suitability for humpback whale (*Megaptera novaeangliae*). White circles indicate cells with one or more animal sightings. Renewable energy areas include a 10 km buffer zone.

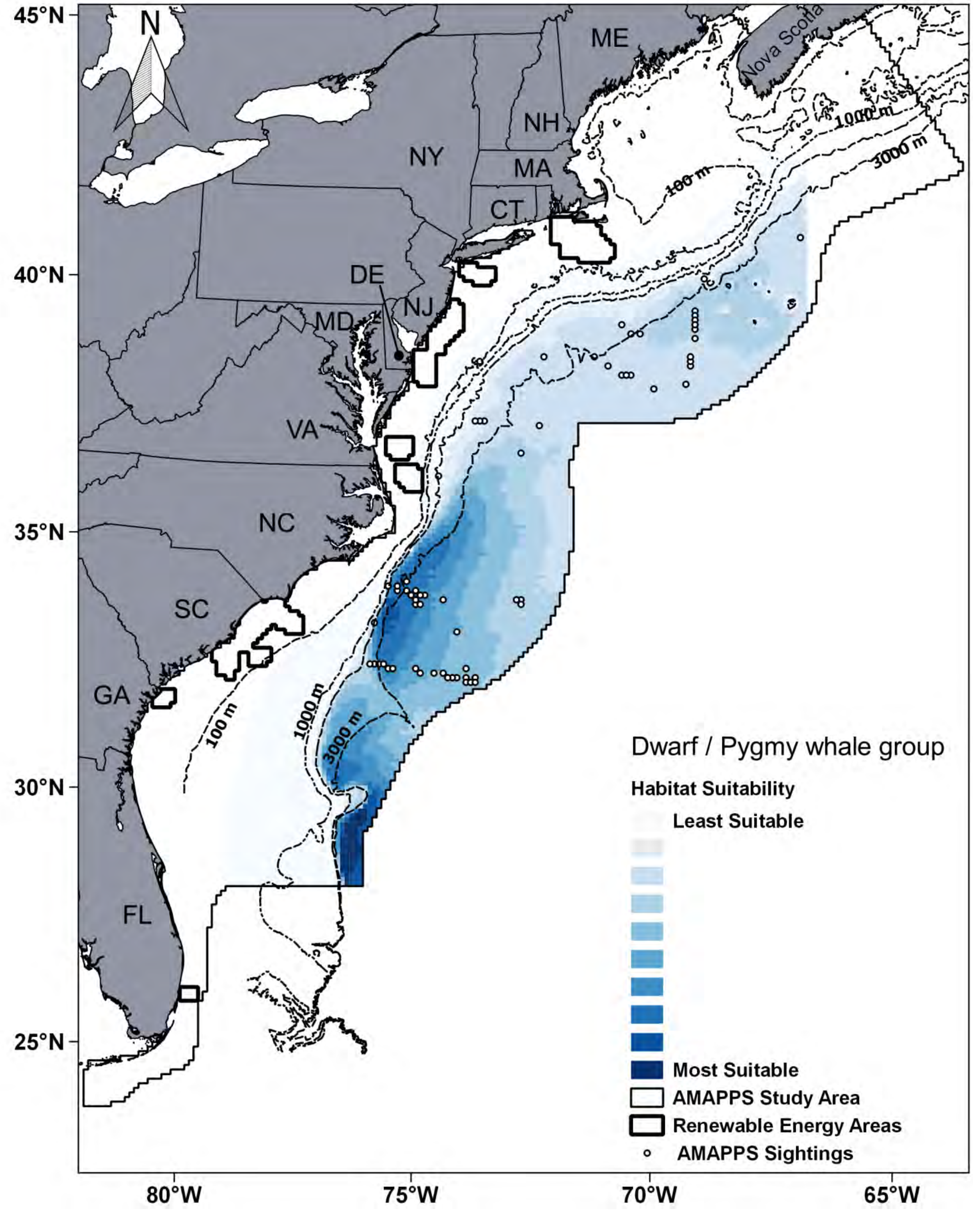

Supplementary Figure S9. Habitat suitability for pygmy/dwarf sperm whale (*Kogia spp*). White circles indicate cells with one or more animal sightings. Renewable energy areas include a 10 km buffer zone.

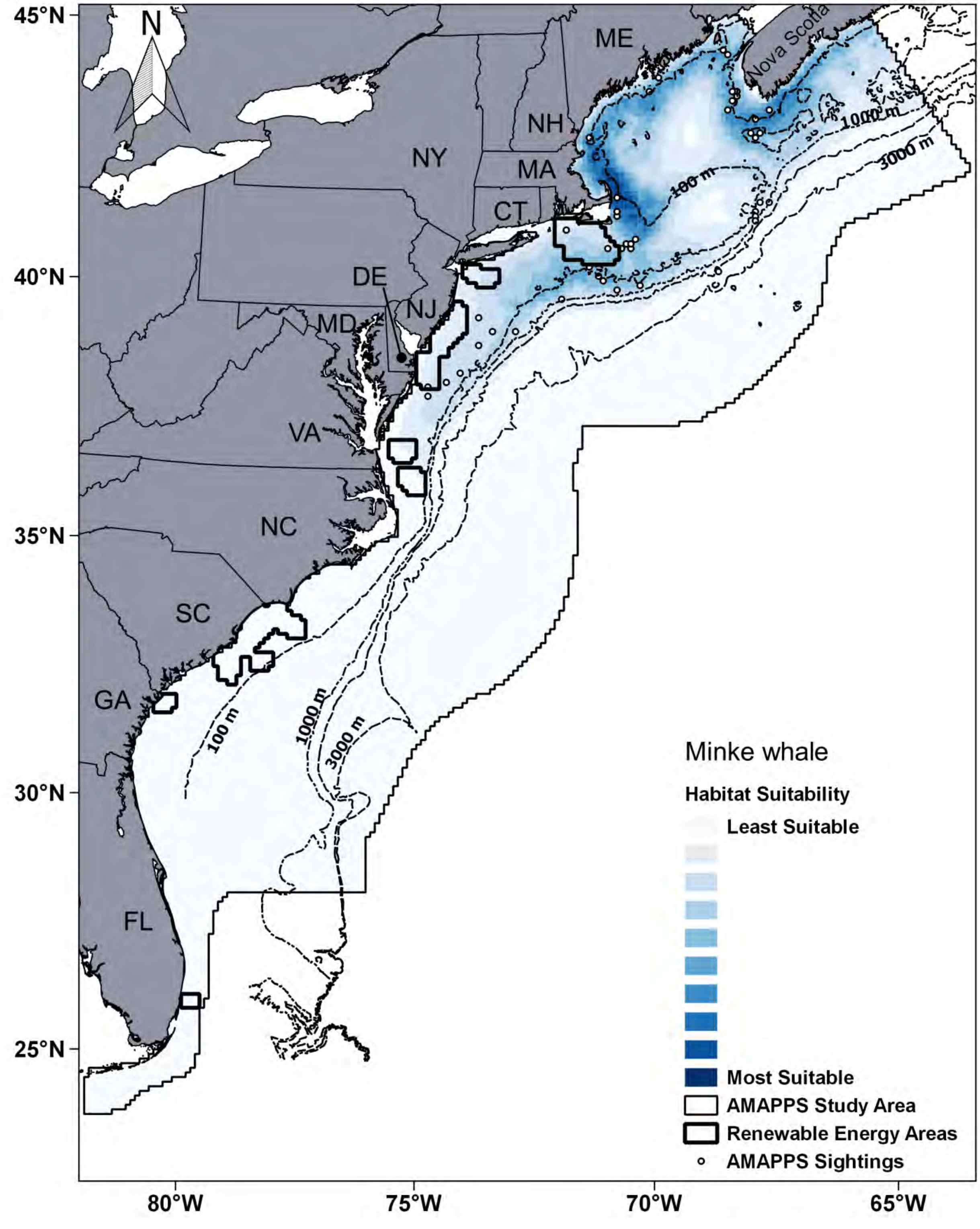

Supplementary Figure S10. Habitat suitability for minke whale (*Balaenoptera acutorostrata*). White circles indicate cells with one or more animal sightings. Renewable energy areas include a 10 km buffer zone.

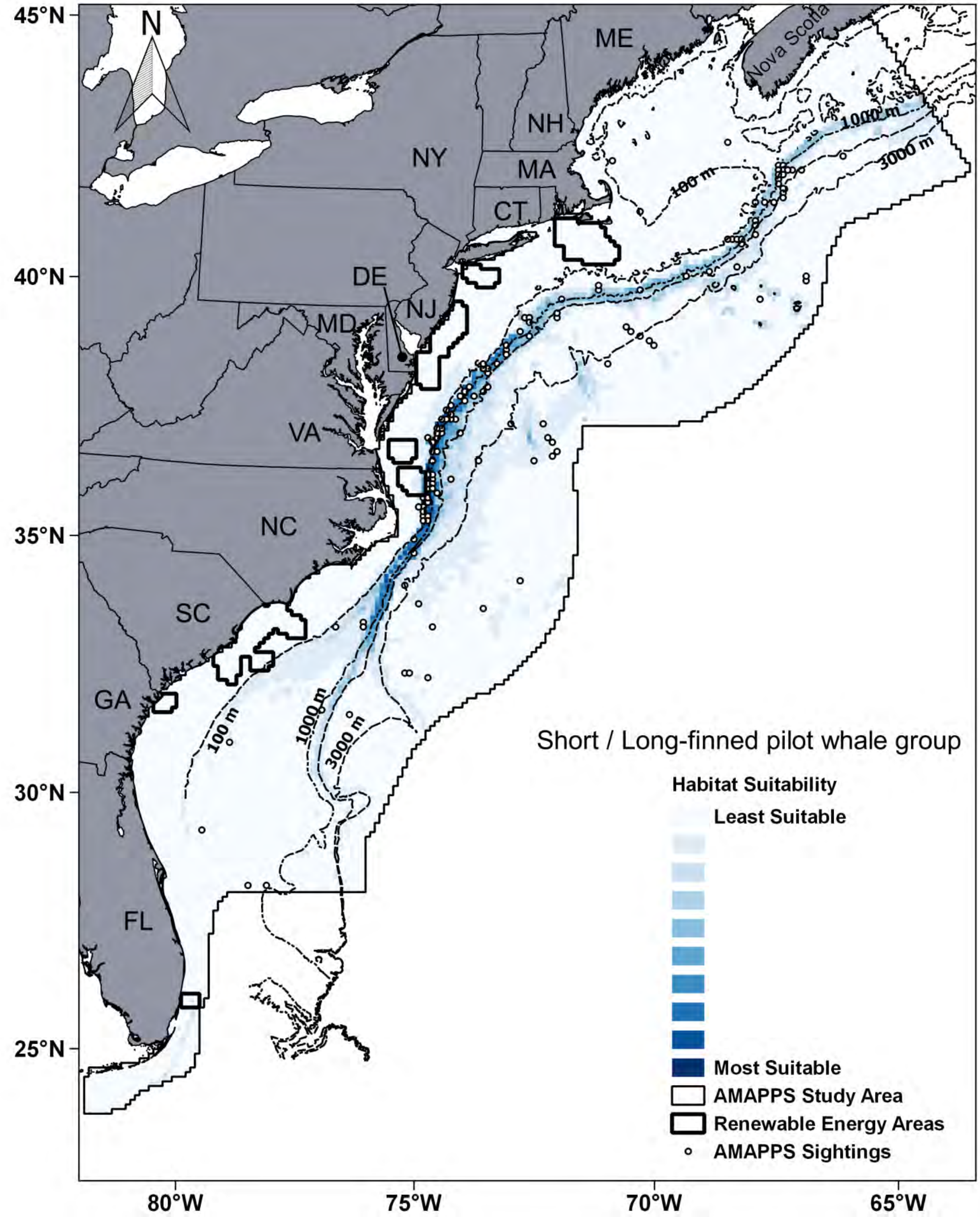

Supplementary Figure S11. Habitat suitability for pilot whale (*Globicephala spp*). White circles indicate cells with one or more animal sightings. Renewable energy areas include a 10 km buffer zone.

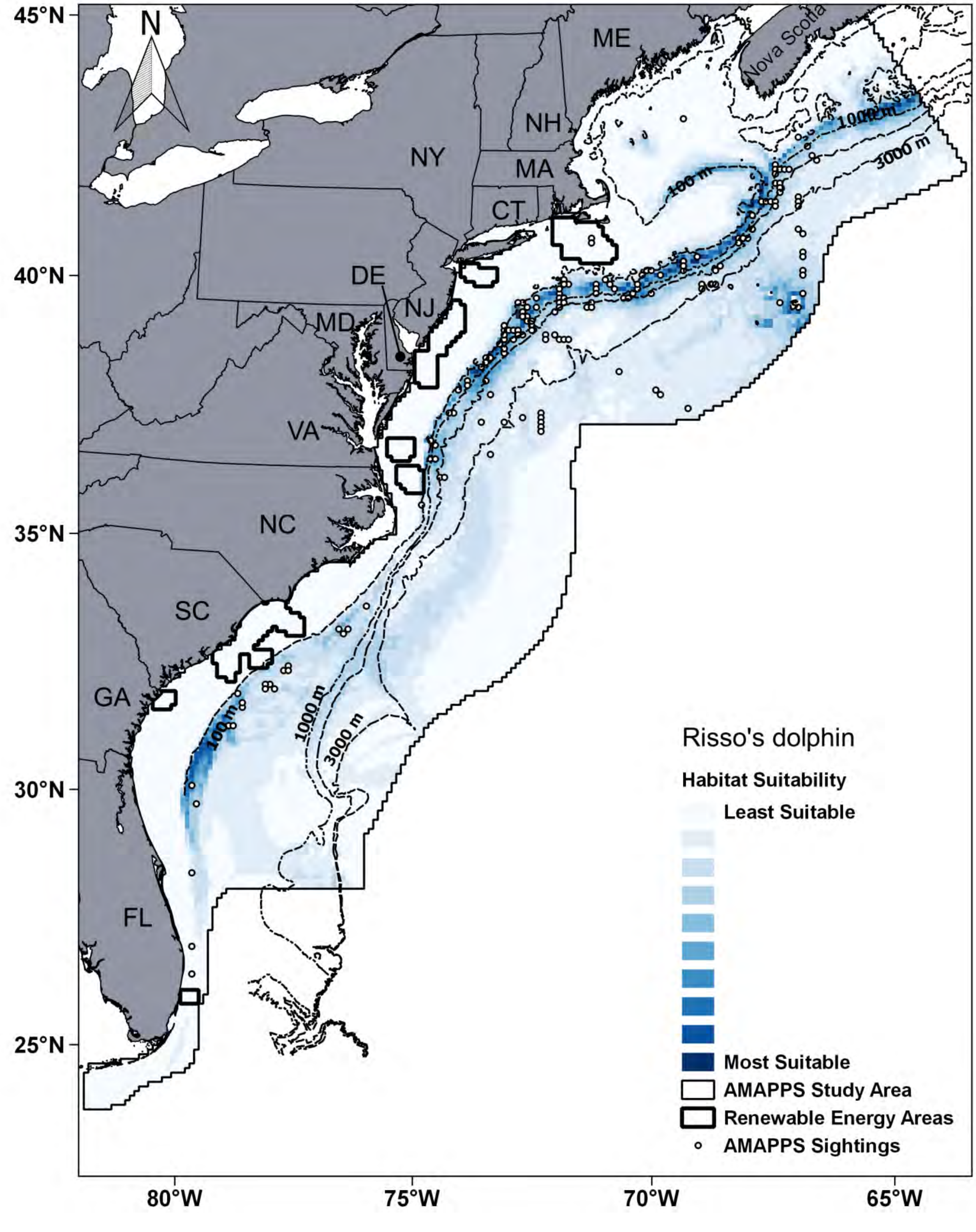

Supplementary Figure S12. Habitat suitability for Risso's dolphin (*Grampus griseus*). White circles indicate cells with one or more animal sightings. Renewable energy areas include a 10 km buffer zone.

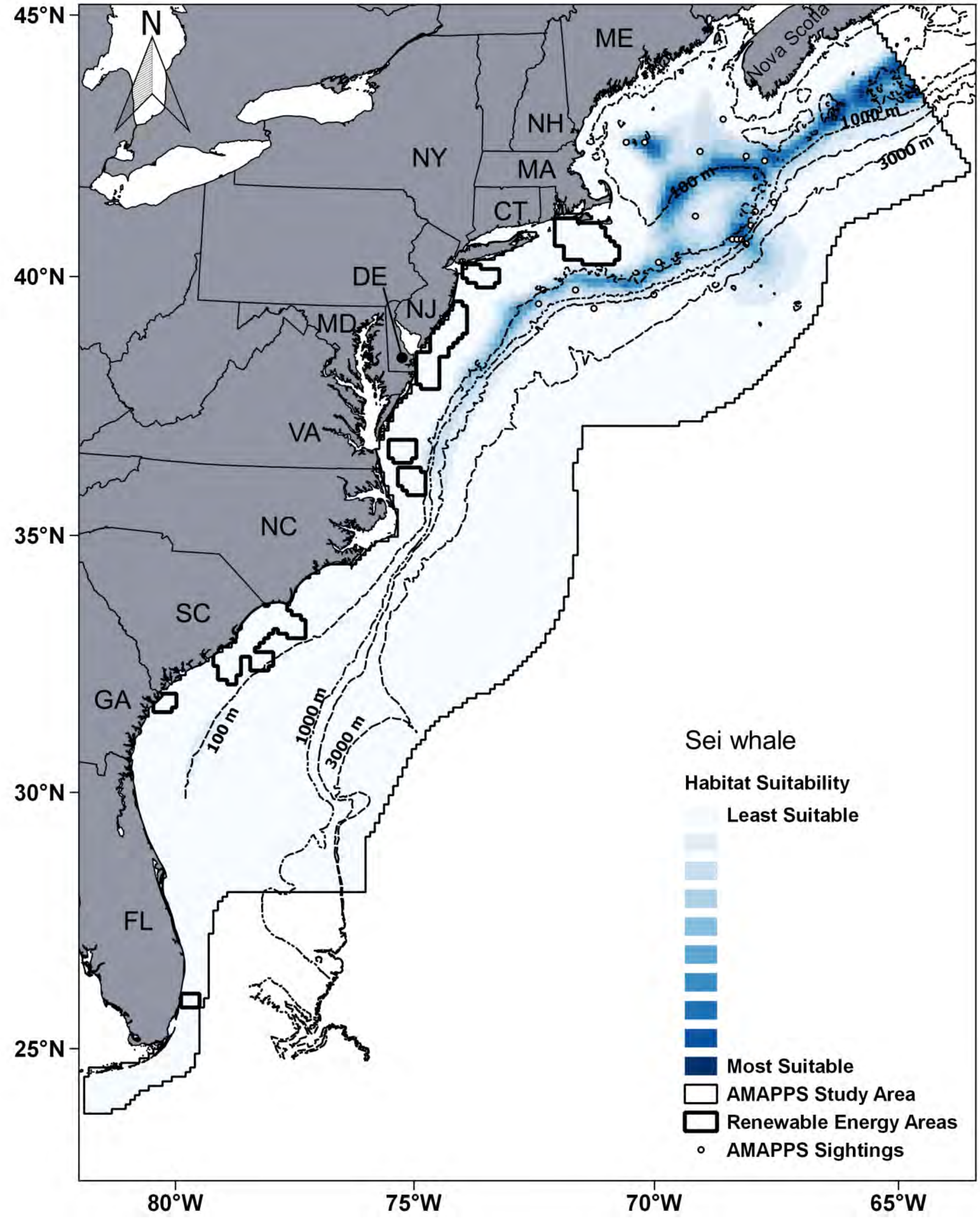

Supplementary Figure S13. Habitat suitability for sei whale (*Balaenoptera borealis*). White circles indicate cells with one or more animal sightings. Renewable energy areas include a 10 km buffer zone.

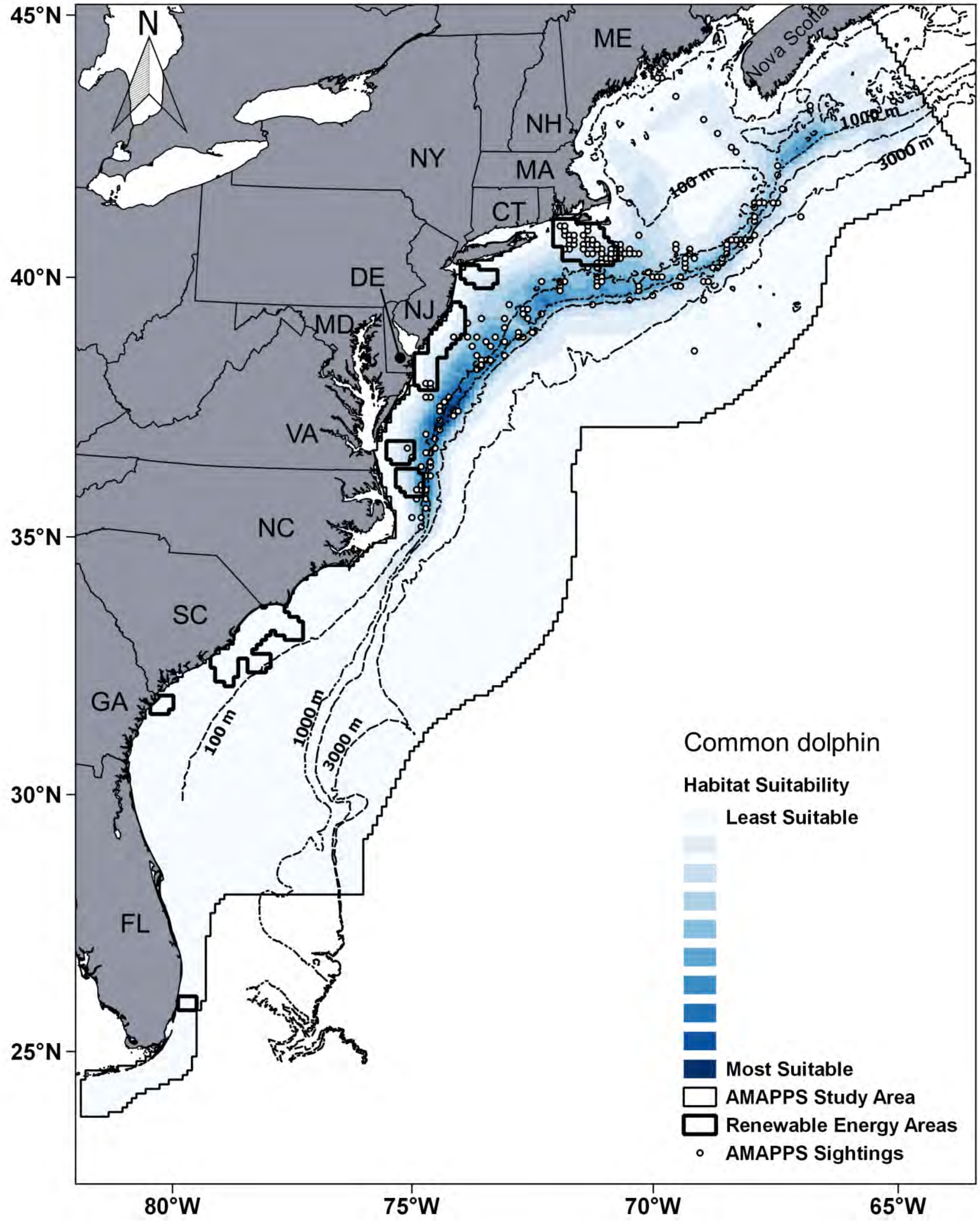

Supplementary Figure S14. Habitat suitability for common dolphin (*Delphinus delphis*). White circles indicate cells with one or more animal sightings. Renewable energy areas include a 10 km buffer zone.

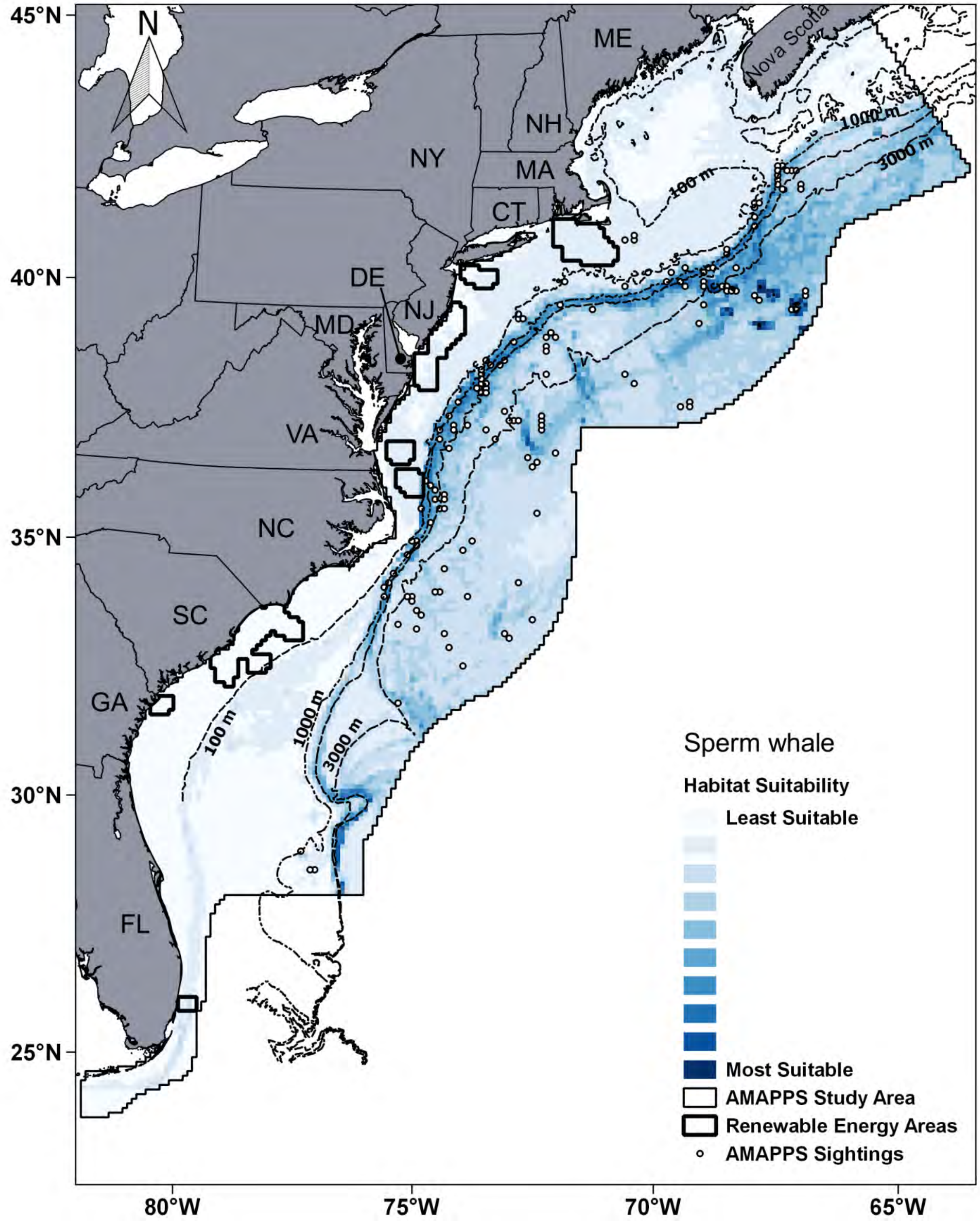

Supplementary Figure S15. Habitat suitability for sperm whale (*Physeter macrocephalus*). White circles indicate cells with one or more animal sightings. Renewable energy areas include a 10 km buffer zone.

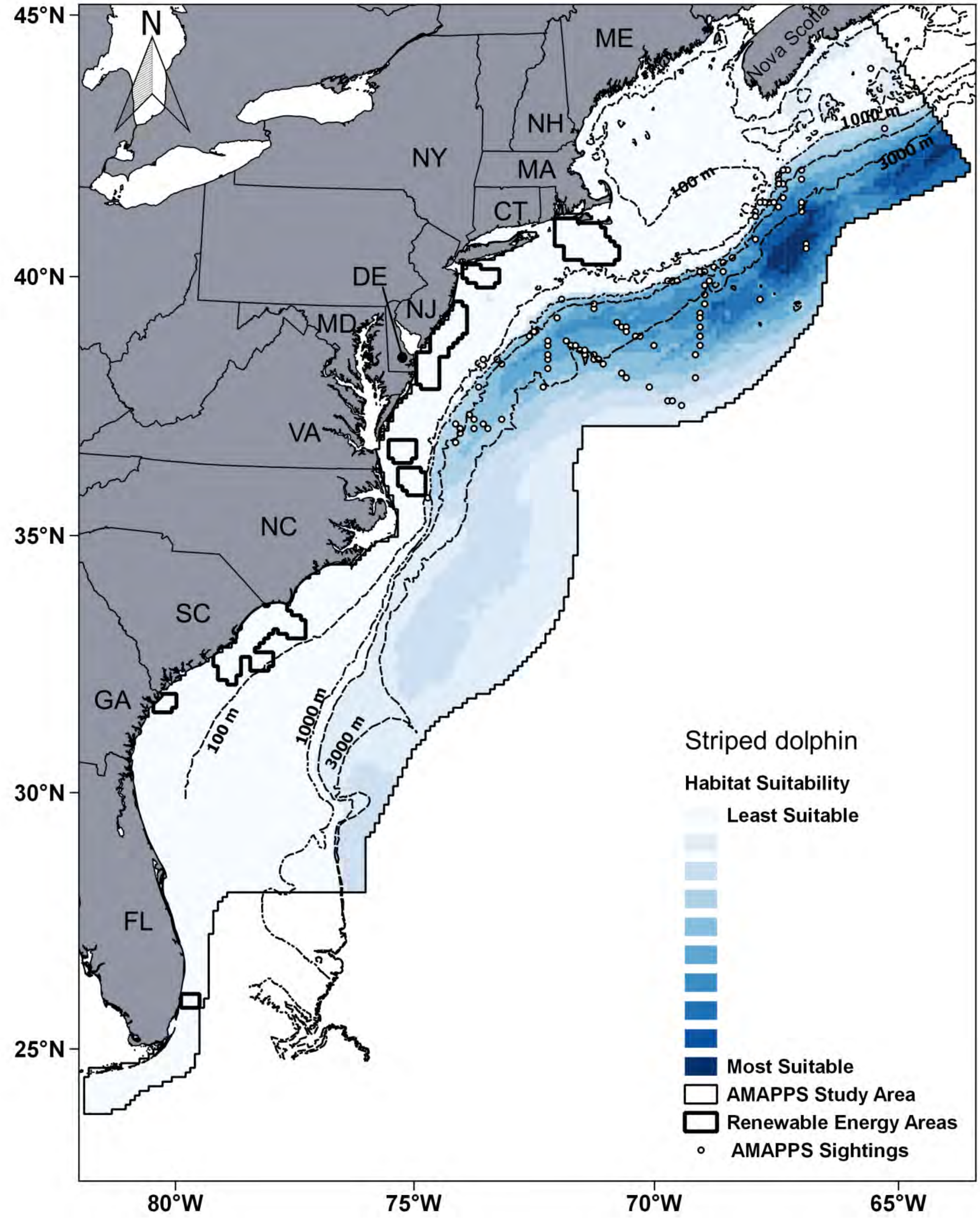

Supplementary Figure S16. Habitat suitability for striped dolphin (*Stenella coeruleoalba*). White circles indicate cells with one or more animal sightings. Renewable energy areas include a 10 km buffer zone.

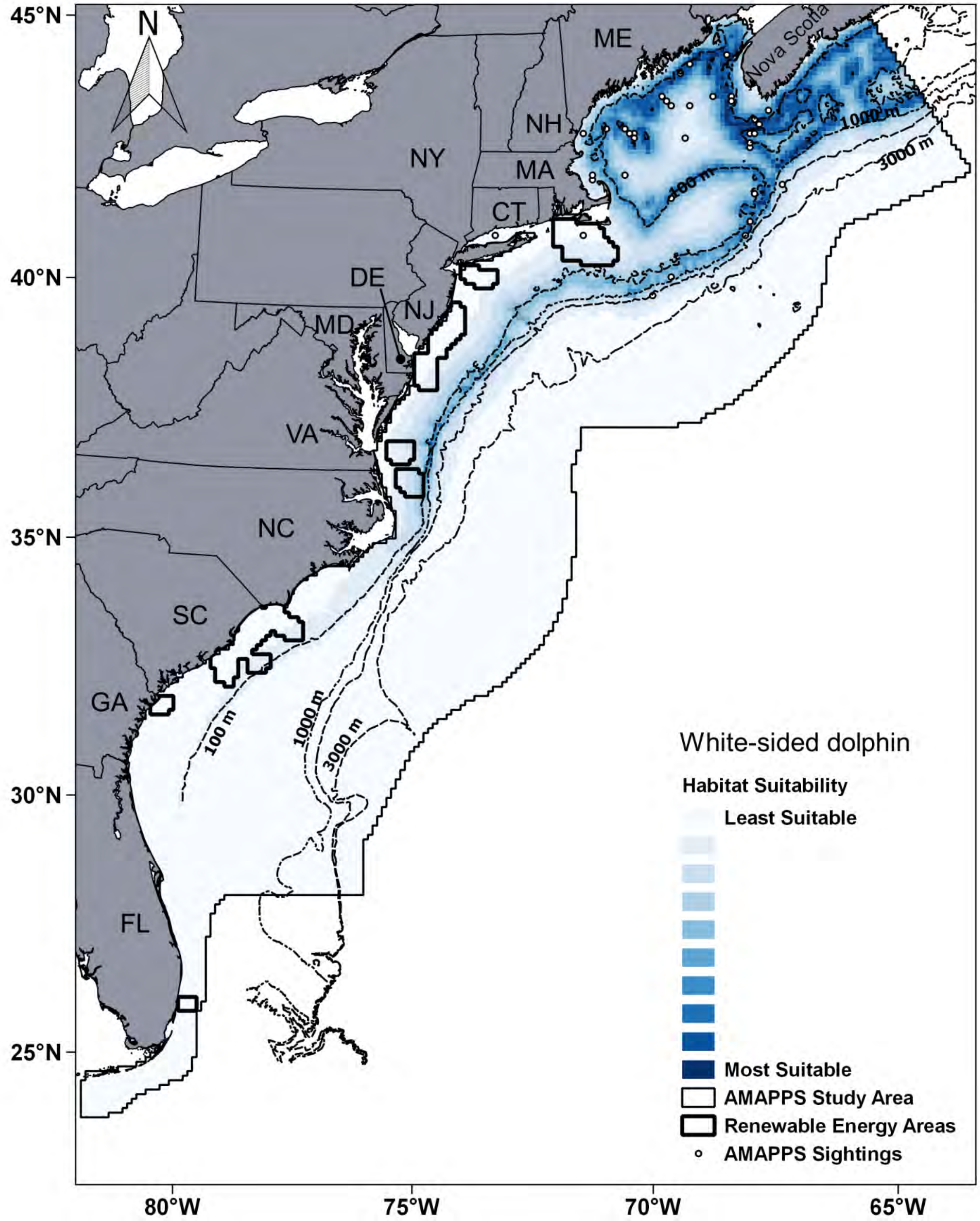

Supplementary Figure S17. Habitat suitability for white-sided dolphin (*Lagenorhynchus acutus*). White circles indicate cells with one or more animal sightings. Renewable energy areas include a 10 km buffer zone.

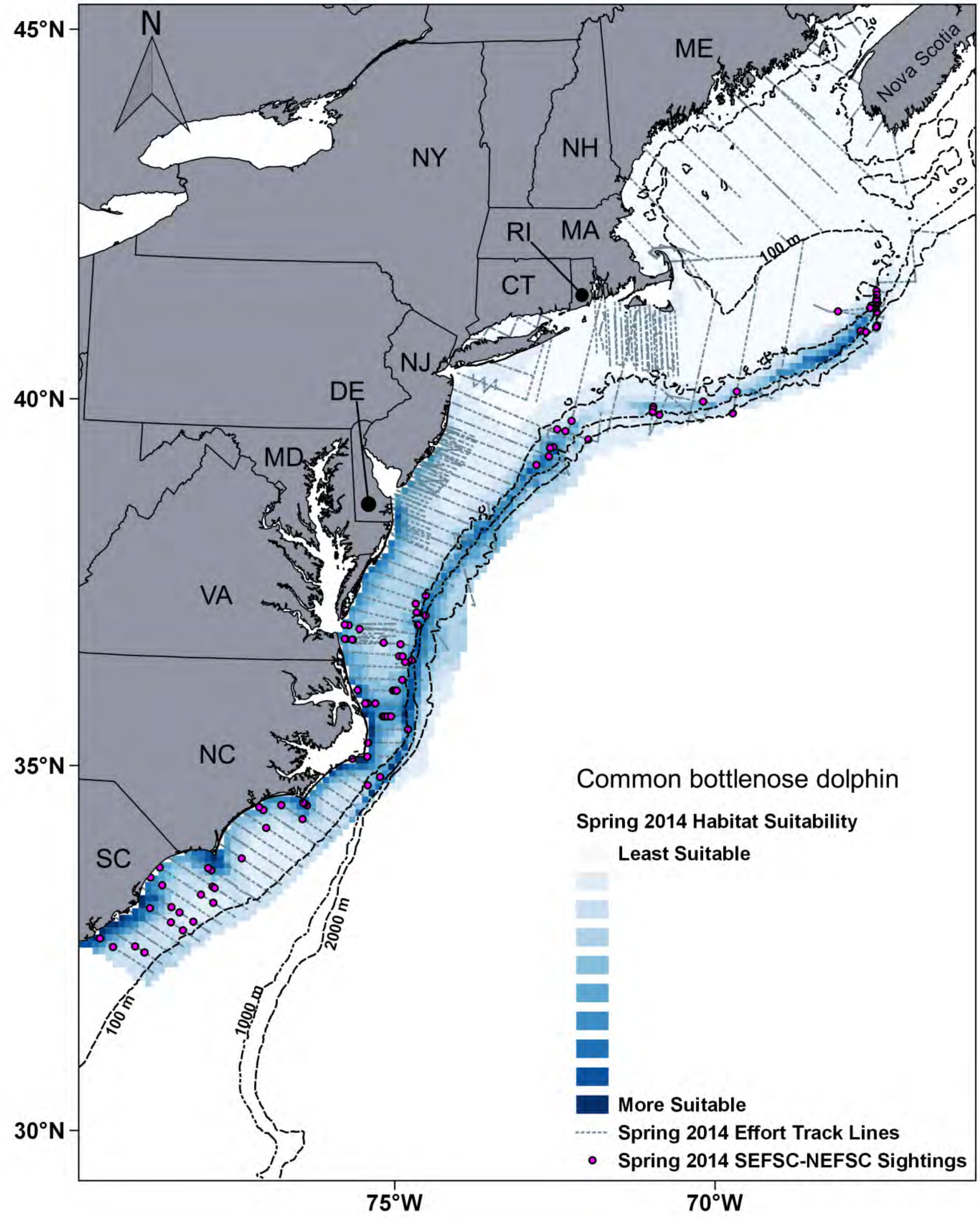

Supplementary Figure S18. Comparison of the common bottlenose dolphin (*Tursiops truncatus*) 2010-13 habitat model definition applied to spring 2014 environmental covariates with the spring 2014 AMAPPS sightings for the species. These sightings were not included in the habitat model development.

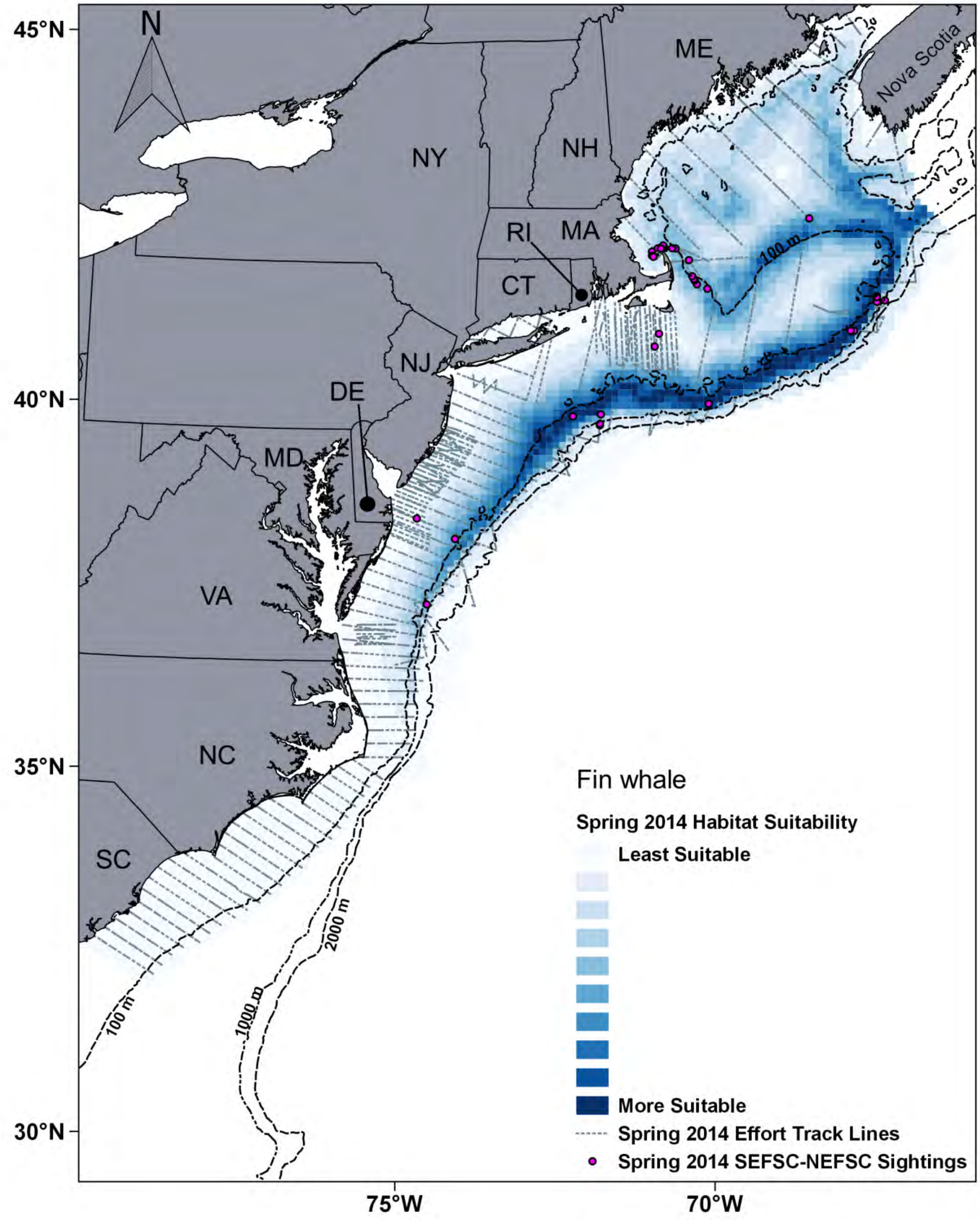

Supplementary Figure S19. Comparison of the fin whale (*Balaenoptera physalus*) 2010-13 habitat model definition applied to spring 2014 environmental covariates with the spring 2014 AMAPPS sightings for the species. These sightings were not included in the habitat model development.

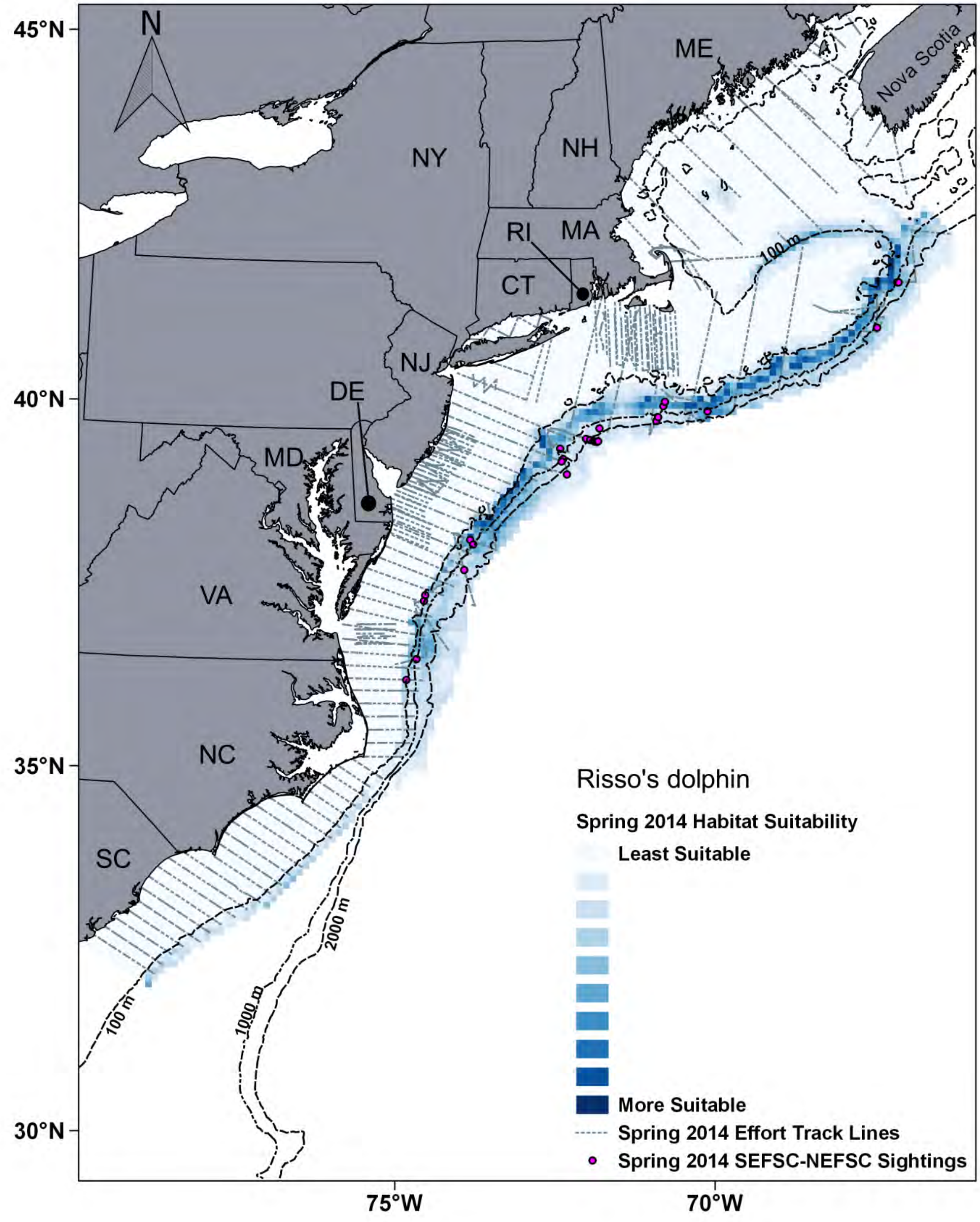

Supplementary Figure S20. Comparison of the Risso's dolphin (*Grampus griseus*) 2010-13 habitat model definition applied to spring 2014 environmental covariates with the spring 2014 AMAPPS sightings for the species. These sightings were not included in the habitat model development.

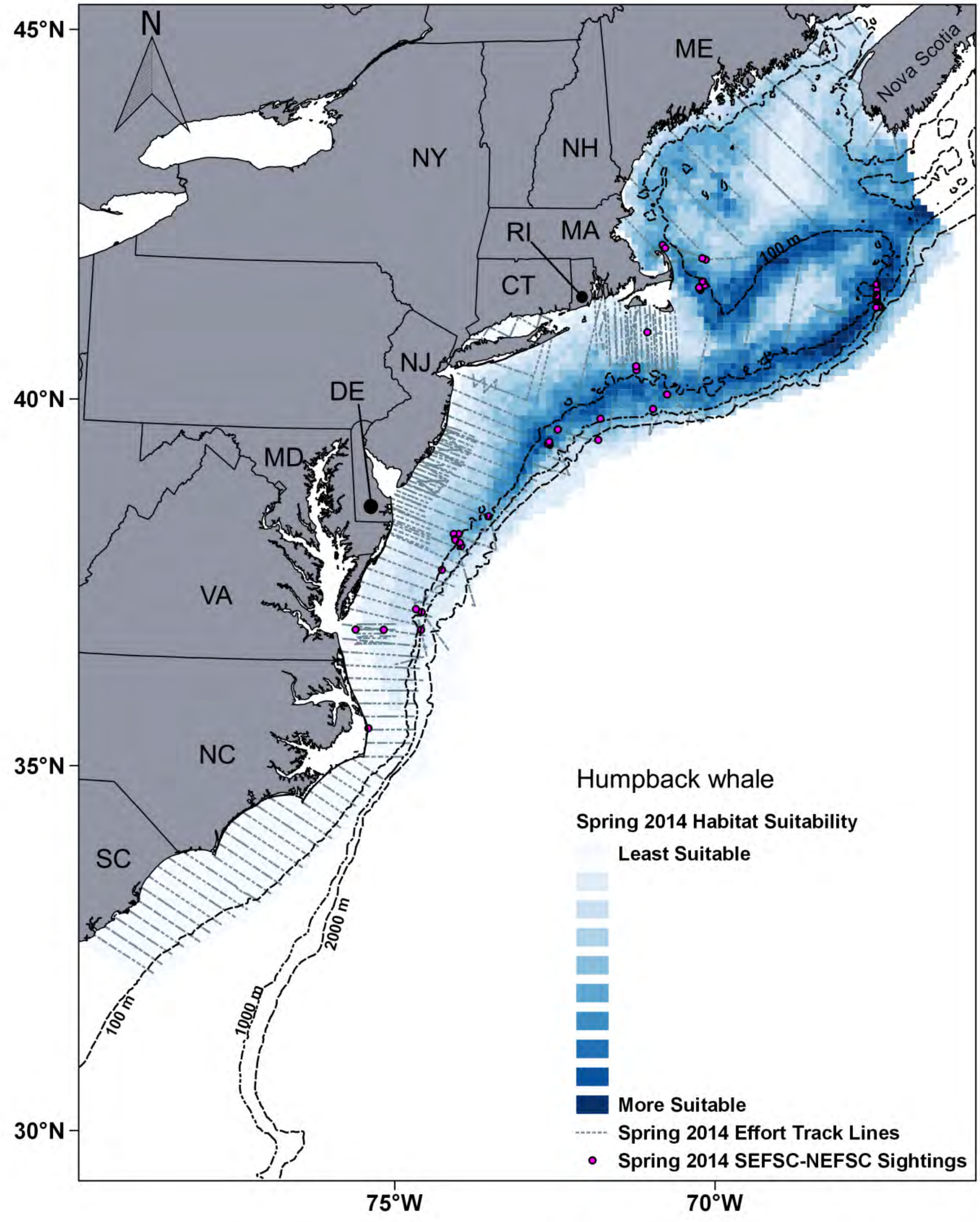

Supplementary Figure S21. Comparison of the humpback whale (*Megaptera novaeangliae*) 2010-13 habitat model definition applied to spring 2014 environmental covariates with the spring 2014 AMAPPS sightings for the species. These sightings were not included in the habitat model development.

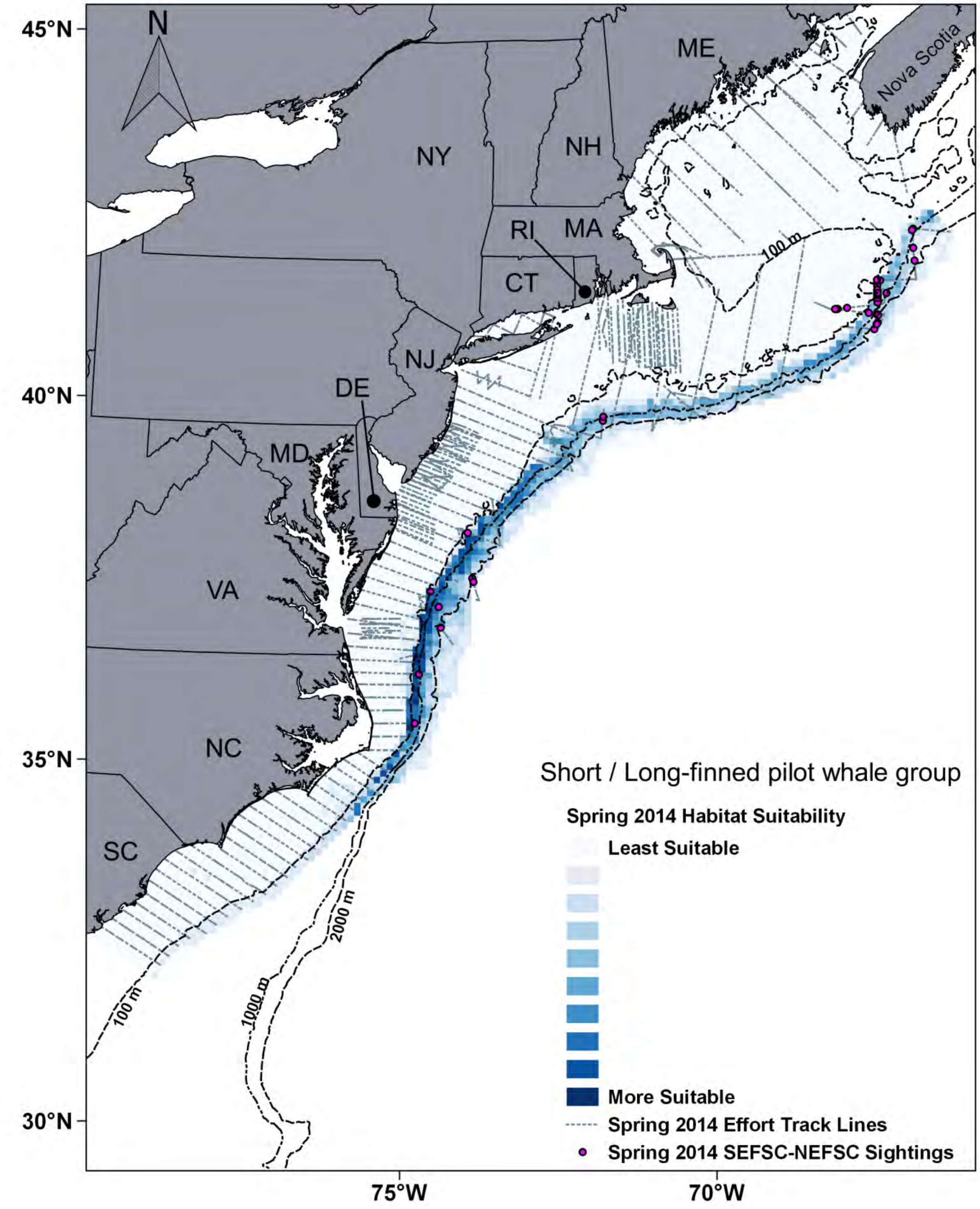

Supplementary Figure S22. Comparison of the pilot whale (*Globicephala* spp) 2010-13 habitat model definition applied to spring 2014 environmental covariates with the spring 2014 AMAPPS sightings for the species. These sightings were not included in the habitat model development.

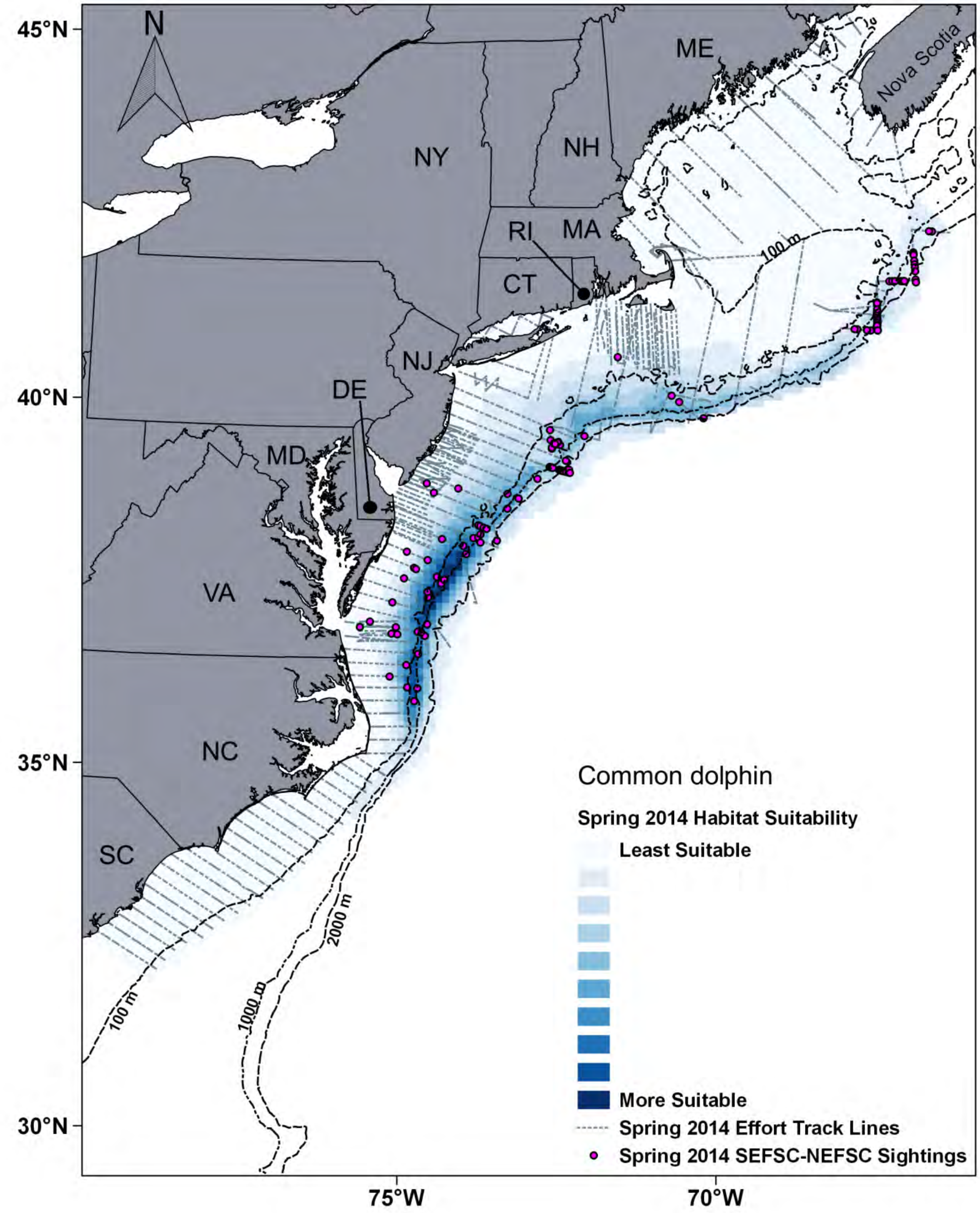

Supplementary Figure S23. Comparison of the common dolphin (*Delphinus delphis*) 2010-13 habitat model definition applied to spring 2014 environmental covariates with the spring 2014 AMAPPS sightings for the species. These sightings were not included in the habitat model development.

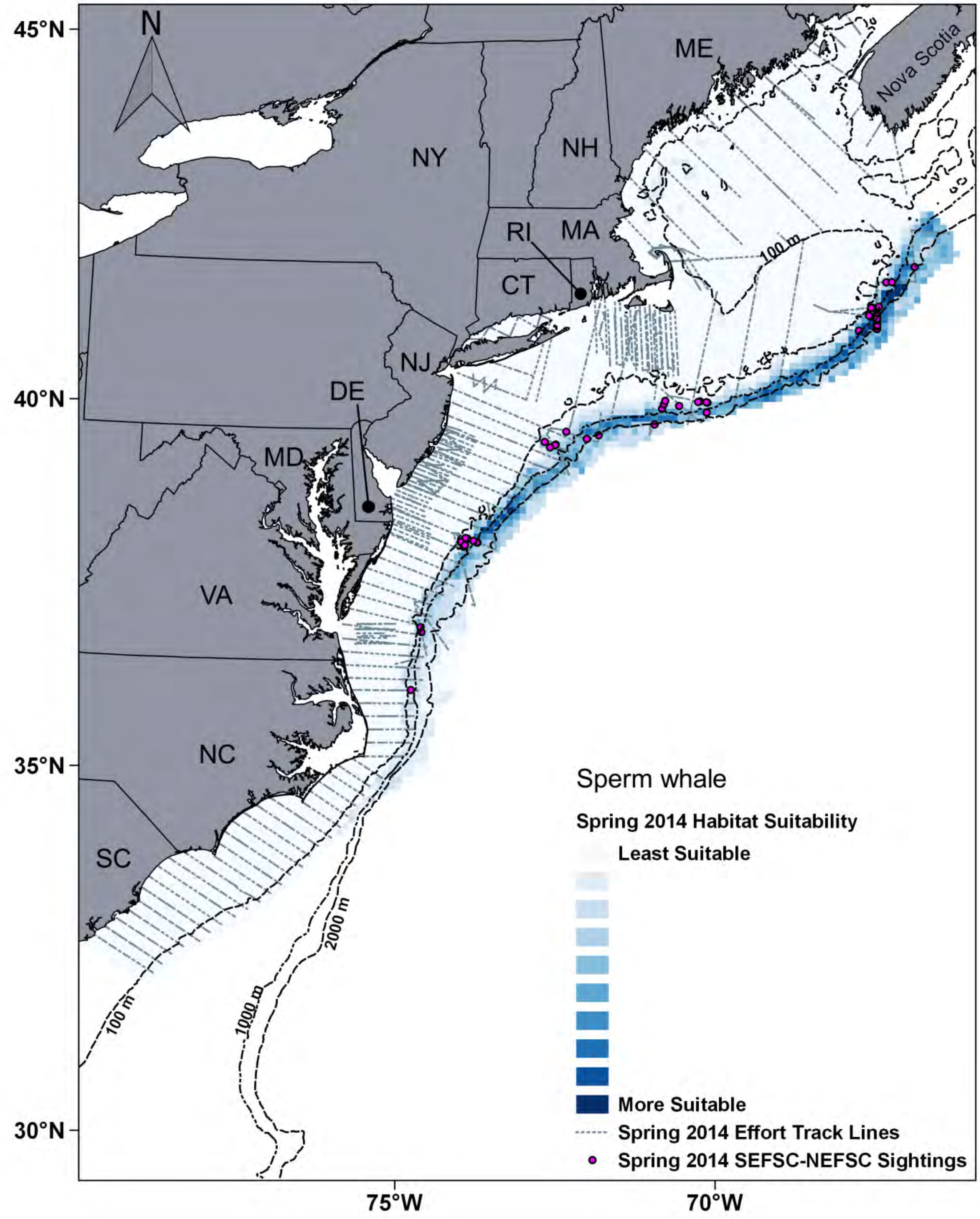

Supplementary Figure S24. Comparison of the sperm whale (Physeter macrocephalus) 2010-13 habitat model definition applied to spring 2014 environmental covariates with the spring 2014 AMAPPS sightings for the species. These sightings were not included in the habitat model development.

REFERENCES

1. Palka, D.L. *et al.* Atlantic Marine Assessment Program for Protected Species: 2010- 2014 (2017). US Dept. of the Interior, Bureau of Ocean Energy Management, Atlantic OCS Region, Washington, DC. OCS Study BOEM 2017-071. Available at: https://www.boem.gov/espis/5/5638.pdf

2. Amante, C. & Eakins, B. W. ETOPO1 1 Arc-Minute Global Relief Model: Procedures, Data Sources and Analysis (2009). NOAA Technical Memorandum NESDIS NGDC-24. National Geophysical Data Center, NOAA. doi:10.7289/V5C8276M [access date: 11/17/14]

3. Simons, R.A. ERDDAP. http://coastwatch.pfeg.noaa.gov/erddap. Monterey, CA: NOAA/NMFS/SWFSC/ERD (2015). [access date: 12/10/14]

4. Chassignet, E.P. *et al.* The HYCOM (Hybrid Coordinate Ocean Model) data assimilative system. *J. Mar. Syst.* **65**, 60–83 (2007). [access date: 11/20/14]

5. AVISO+. The Ssalto/*Duacs* altimeter products were produced and distributed by the Copernicus Marine and Environment Monitoring Service (CMEMS) (http://www.marine.copernicus.eu) [access date: 11/20/14]
